# Diverse Routes toward Early Somites in the Mouse Embryo

**DOI:** 10.1016/j.devcel.2020.11.013

**Published:** 2021-01-11

**Authors:** Carolina Guibentif, Jonathan A. Griffiths, Ivan Imaz-Rosshandler, Shila Ghazanfar, Jennifer Nichols, Valerie Wilson, Berthold Göttgens, John C. Marioni

**Affiliations:** 1 Department of Haematology, University of Cambridge, CB2 0AW Cambridge, UK; 2 Wellcome-Medical Research Council Cambridge Stem Cell Institute, University of Cambridge, CB2 0AW Cambridge, UK; 3 Sahlgrenska Center for Cancer Research, Department of Microbiology and Immunology, University of Gothenburg, 413 90 Gothenburg, Sweden; 4 Cancer Research UK Cambridge Institute, University of Cambridge, CB2 0RE Cambridge, UK; 5 Centre for Regenerative Medicine, Institute for Stem Cell Research, School of the Biological Sciences, University of Edinburgh, EH16 4UU Edinburgh, UK; 6 Department of Physiology, Development and Neuroscience, University of Cambridge, CB2 3DY Cambridge, UK; 7 Wellcome Sanger Institute, Wellcome Genome Campus, CB10 1SA Cambridge, UK; 8 European Molecular Biology Laboratory, European Bioinformatics Institute, European Molecular Biology Laboratory, EBI), Wellcome Genome Campus, CB10 1SD Cambridge, UK

**Keywords:** developmental trajectories, cell fate regulation, neuromesodermal progenitors, Brachyury, somites

## Abstract

Somite formation is foundational to creating the vertebrate segmental body plan. Here, we describe three transcriptional trajectories toward somite formation in the early mouse embryo. Precursors of the anterior-most somites ingress through the primitive streak before E7 and migrate anteriorly by E7.5, while a second wave of more posterior somites develops in the vicinity of the streak. Finally, neuromesodermal progenitors (NMPs) are set aside for subsequent trunk somitogenesis. Single-cell profiling of *T*^−/−^ chimeric embryos shows that the anterior somites develop in the absence of T and suggests a cell-autonomous function of T as a gatekeeper between paraxial mesoderm production and the building of the NMP pool. Moreover, we identify putative regulators of early T-independent somites and challenge the T-Sox2 cross-antagonism model in early NMPs. Our study highlights the concept of molecular flexibility during early cell-type specification, with broad relevance for pluripotent stem cell differentiation and disease modeling.

## Highlights

• Multiple transcriptional trajectories underlie somite emergence
• Outlining the *in vivo* molecular journey toward the anterior T-independent somites
• T does not negatively regulate Sox2 to control neural output at E8.5

## Graphical Abstract

**Figure:**
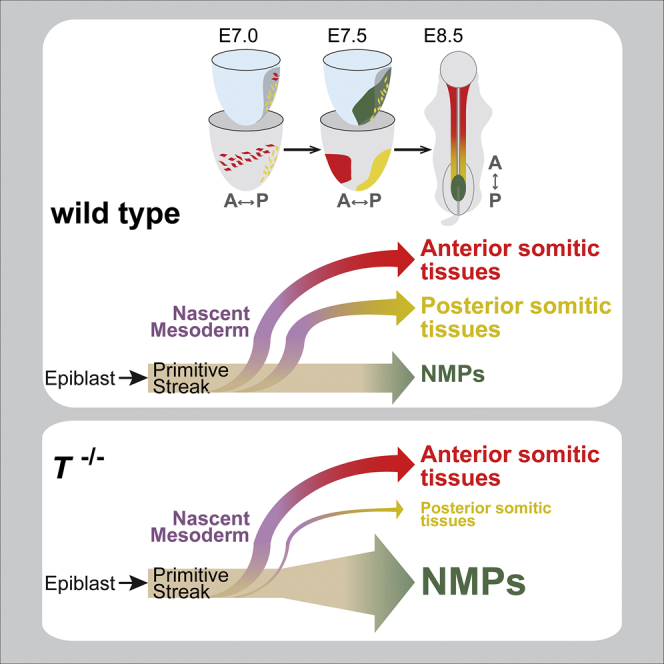

## Teaser

Guibentif, Griffiths et al. reconstruct three transcriptional journeys from the pluripotent mouse epiblast to anterior somites, posterior somites, and neuromesodermal progenitors (NMPs) present at embryonic day 8.5. Single-cell profiling of *T*^−/−^↔WT chimeras validates the molecular journey toward T-independent anterior somites and challenges the notion that NMP differentiation is regulated by T and Sox2 mutual antagonism.

## Introduction

The recent emergence of high throughput single-cell RNA-sequencing (scRNA-seq) assays has allowed researchers to survey entire transcriptional landscapes of development in numerous species (Cao et al., 2019; Packer et al., 2019; Pijuan-Sala et al., 2019; Wagner et al., 2018). Somites are transient segments of the paraxial mesoderm that give rise to the axial skeleton and associated musculature. Following formation of the most anterior somites, subsequent axis elongation is fueled by a pool of neuromesodermal progenitors (NMPs), which give rise to neural components of the spinal cord as well as the mesodermal tissue of the somites (Pourquié, 2001; Tzouanacou et al., 2009). NMPs are characterized by co-expression of transcription factors associated with gastrulation, mesodermal, and neural development, including *Brachyury* (*T*), *Sox2,* and *Nkx1-2* (Henrique et al., 2015; Steventon and Martinez Arias, 2017; Wilson et al., 2009).

Starting as uniform blocks of epithelium, somites compartmentalize into ventral sclerotome (which gives rise to major elements of the skeleton, such as the vertebrae and ribs) and dorsal dermomyotome (precursor of skeletal muscles and of the skin of the back; Keynes and Stern, 1988). Somitogenesis is often portrayed as a relatively uniform process, regulated by an interacting network of signaling pathways and transcription factors such as Fgf, Wnt, Notch, T, and Tbx6 (Chapman and Papaioannou, 1998; Hubaud and Pourquié, 2014; Martin and Kimelman, 2008). However, multiple lines of evidence indicate that disruption of these canonical somite regulators has little effect on the formation of the first, most anterior, somites both in mouse and in fish (Nowotschin et al., 2012; Pourquié, 2001), and the molecular programs responsible for the formation of these occipital somites remain poorly defined. Occipital somites differentiate early in development and do not give rise to repetitive skeletal structures. In chick, gene-expression analysis has demonstrated a specific molecular make-up of the anterior-most somites (Rodrigues et al., 2006), and in Amphioxus, there are at least three distinct transcriptional networks regulating the emergence of specific anterior-posterior somite subsets (Aldea et al., 2019). Overall, these observations suggest that multiple, potentially independent, molecular pathways can generate somites.

Here, we used trajectory inference in a transcriptional atlas of mouse gastrulation, as well as single-cell profiling of *T*^−/−^ embryonic chimeras, to show that somitic tissues present in the E8.5 mouse embryo emerge through different developmental pathways. A first wave arises from early progenitors ingressing through the primitive streak before E7.0 and migrating anteriorly before E7.5, while a second wave of more posterior somitic progenitors remains posteriorly in the streak region. At E7.5, precursors of both waves are anatomically segregated, express different levels of *T* and *Tbx6*, and are exposed to distinct signaling environments. Nevertheless, both will activate the “core” somitic transcriptional program characterized by upregulation of *Tcf15* and *Meox1*. The presence of two distinct waves is corroborated in *T*^−/−^ chimeric embryos, where *T*^−/−^ cells contribute normally to the first wave but are highly depleted in the second wave. This depletion is accompanied by increased contribution to a third developmental trajectory, leading from epiblast to E8.5 NMPs, suggesting that T may function as a gatekeeper regulating the allocation of streak cells to the NMP pool. Finally, we provide evidence that in E8.5 NMPs, T acts predominantly as a transcriptional activator and may not be necessary for *Sox2* repression.

## Results

### The E8.5 Mouse Embryo Contains Somitic Cells with Distinct Transcriptional Signatures

A previously published reference atlas of mouse gastrulation reported 37 major cell types (Pijuan-Sala et al., 2019). To characterize the heterogeneity of E8.5 paraxial mesoderm, we subclustered cells belonging to the somitic and paraxial mesoderm clusters (Figures S1A–S1E). Presomitic mesoderm was identified by expression of *Fgf17*, *Tbx6*, *Cyp26a1*, *T*, *Hes7*, *Dll3*, *Lef1*, *Rspo3*, and *Dkk1* (Bessho et al., 2001; Cao et al., 2004; Chal et al., 2015; Chapman et al., 1996; Galceran et al., 2004; Sakai et al., 2001; Takahashi et al., 2003; Wahl et al., 2007) and cranial mesoderm by elevated levels of *Tbx1*, *Foxl2*, and *Pitx2* (Dastjerdi et al., 2007; Marongiu et al., 2015; Nandkishore et al., 2018; Sambasivan et al., 2011; Shih et al., 2007). Four somitic subclusters included two sets of uncompartmentalized somitic cells (co-expressing *Tcf15* and *Meox1*) (Burgess et al., 1996; Mankoo et al., 2003) separated by clusters indicating commitment to sclerotome (*Pax1* and *Pax9*) (Peters et al., 1999) and dermomyotome (*Dmrt2*, *Pax3*, and *Meox2*) (Kassar-Duchossoy et al., 2005; Sato et al., 2010) (Figure S1E).

**Figure 1:**
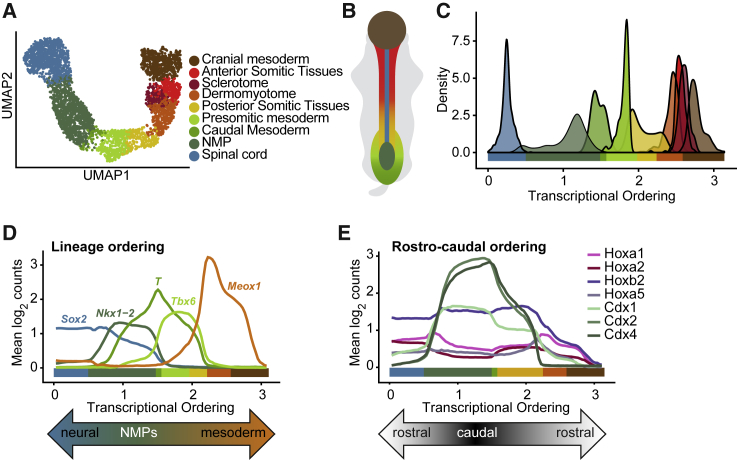
Two Distinct Transcriptional Subsets of Somites at E8.5 (A) Uniform manifold approximation and projection (UMAP) representation of the axial elongation-related tissues present at E8.5. (B) Schematic of the axial elongation-related tissues in the anatomy of the E8.5 mouse embryo. For color code, refer to legend in (A). (C) Distribution of E8.5 axial elongation-related tissues along one-dimensional transcriptional ordering. For color code, refer to legend in (A). (D) Marker expression along one-dimensional transcriptional ordering delimits neural and paraxial cell types, including bipotent NMPs. Expression levels are shown as the mean of the expression values in a sliding window of width 10% of the length of the ordering. (E) Homeobox genes provide rostrocaudal orientation of diffusion pseudotime ordering with bipotent NMPs in the center of the ordering, corresponding to the caudal end of the embryo, and neural and paraxial cell types at the edges, expressing rostral Hox genes. Expression levels are shown as in (D). See also Figure S1.

We next investigated the transcriptional similarity between these populations and other cell types related to axial elongation at E8.5—NMPs and spinal cord. Diffusion processes revealed a one-dimensional ordering (Figures 1A–1C) consistent with higher-dimensional representations (Figure S1F), from *Sox2*-expressing spinal cord, through NMPs co-expressing *Sox2*, *T*, and *Nkx1-2*, to *Meox1*-expressing paraxial lineages (Figure 1D). Homeobox transcription factor expression supported an underlying spatial component to this ordering, with caudal Cdx genes peaking in the center, at the position of NMPs (Figures 1E and S1G). The cranial mesoderm signature is present at the rostral-most, paraxial end of the ordering (Figures 1E, S1E and S1G). Next in the ordering are somitic cells flanked by dermomyotome and sclerotome clusters. With this signature of ongoing compartmentalization, these represent the most developed and, therefore, the most anterior somites. They are followed by uncompartmentalized, less mature, and more posterior somitic cells, followed by presomitic mesoderm and finally NMPs, in the more posterior region of the E8.5 embryo (Figures 1B and 1C).

### Inference of Distinct Developmental Routes for E8.5 Somitic Tissues

**Figure 2:**
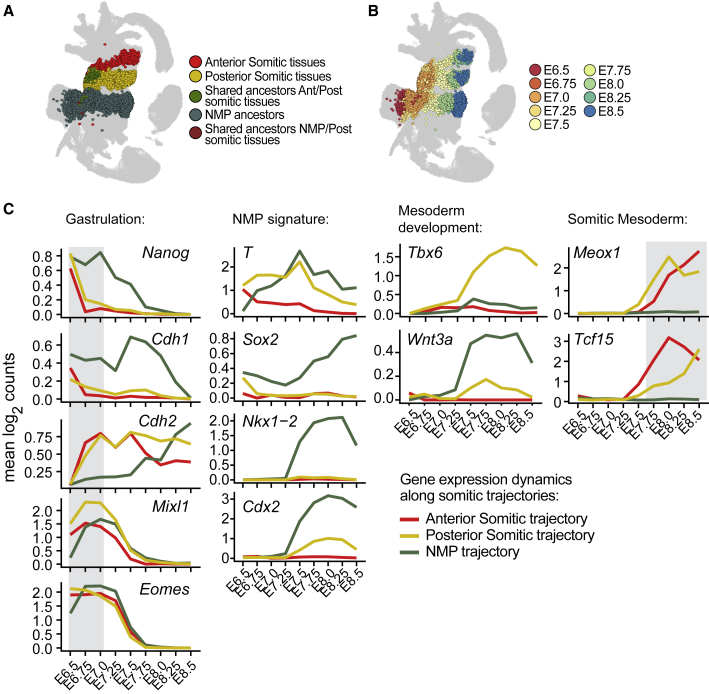
Identification of Distinct Developmental Trajectories toward NMPs and Anterior and Posterior Somitic-Cell Subsets (A) UMAP layout from Pijuan-Sala et al. (2019) highlighting cells belonging to the developmental trajectories for anterior somitic tissues, the newly formed posterior somitic tissues, and NMPs present at E8.5, predicted using WOT analysis. For visualization purposes, the rare populations of shared ancestors were plotted on top. (B) UMAP layout from Pijuan-Sala et al. (2019) highlighting the same cells as in (A) colored by sampling time point. (C) Gene-expression dynamics along the three developmental trajectories reveals distinct transcriptional programs. y axis: mean log_2_ (normalised counts). See also STAR Methods; Figure S2; Table S1.

Having characterized two transcriptionally distinct anterior and posterior sets of somitic cells, as well as a caudal NMP pool at E8.5, we next investigated their putative developmental origins. We reconstructed developmental trajectories using an optimal transport approach (WOT) (Schiebinger et al., 2019; STAR Methods; Figures 2A and 2B), which assigns “mass” to each cell at the clusters featuring the presumed trajectory endpoints and then transfers that mass sequentially backward between cells in adjacent time points that are transcriptionally similar. For each cell, the “mass” for each of the three endpoints allowed us to allocate it to a given trajectory based on the highest mass contribution. As such, WOT enables incorporation of real-time information of the 9 sequential time points from E6.5 to E8.5 covered in the reference atlas; the classification of cells along a trajectory is, thus, not only based on their transcriptional similarity but also on time-point progression. NMPs could be traced back to the epiblast at E6.5, while both somitic trajectories originate from E6.5 primitive streak cells. Separation between anterior and posterior somitic trajectories occurred within E7.0-E7.5 nascent mesoderm (Figures 2B and S2A), suggesting that the diversification of these two populations occurs following ingression through the streak.

Consistent with reported features of gastrulation, both anterior and posterior somitic trajectories display a sharp early downregulation of *Nanog* coupled with a shift in cadherin expression (*Cdh1* to *Cdh2*), which is characteristic of epithelial-to-mesenchymal transition (EMT) (Morgani et al., 2018). By contrast, for the NMP trajectory, these processes occur gradually after E7.0 (Figure 2C). Expression of NMP markers over time confirms the known NMP signature, with expression of *T*, *Sox2*, *Nkx1-2*, and *Cdx2* at E7.5 being maintained up to E8.5. In this trajectory, the persistence of *Cdh1* expression throughout upregulation of *Cdh2* between E7.0 and E8.25 is consistent with an “incomplete” EMT in NMPs (Dias et al., 2020). Inspection of additional EMT genes, including *Epcam* (epithelial marker) and *Vim* (mesenchymal marker), reinforced this notion, with co-expression detected in half of the predicted NMP ancestors between E7.5 and E8.0 (Figures S2B–S2D).

Expression of early mesoderm markers, *Eomes* and *Mixl1*, in all three trajectories is followed by upregulation of the somite markers, *Meox1* and *Tcf15*, specifically in the two somitic trajectories. These two trajectories showed clear molecular divergence at E7.5 (before upregulation of *Meox1* and *Tcf15*), with upregulation of *Wnt3a*, *T*, and *Tbx6* specific to the posterior trajectory (Figure 2C).

In addition to examining known regulators, we performed unbiased pair-wise comparisons of gene expression along the entire length of the three trajectories. We examined for each gene whether its expression pattern significantly differed between each pair-wise combination of trajectories, using as input data the mean expression level of each trajectory at each time point (see STAR Methods; Table S1). Gene set enrichment analysis using the Molecular Signatures Database Hallmark gene set collection (Liberzon et al., 2015; Subramanian et al., 2005) revealed that genes displaying distinct behaviors between the three trajectories were enriched for the EMT process (Figure S2E), consistent with our targeted analysis (Figures S2B–S2D). The process of myogenesis was enriched in the anterior versus posterior somitic trajectories comparison, likely due to the different maturation kinetics of these two sets of somites, reflected by the dynamics of the myogenesis regulator *Mef2c* (Figures S2E and S2F). The mTORC1 pathway was also enriched, with distinct expression of the upstream regulator *Pdk1* and of the downstream targets *Slc2a1* and *Slc2a3* (Figures S2E and S2F). Differences between anterior and posterior somitogenesis have been noted previously (Nowotschin et al., 2012; Rashbass et al., 1991). This inferred transcriptional trajectory leading from the primitive streak to anterior somitic tissues now provides an unbiased molecular description of this process.

### Canonical Regulators of Somitogenesis Are Depleted in the Anterior Trajectory

**Figure 3:**
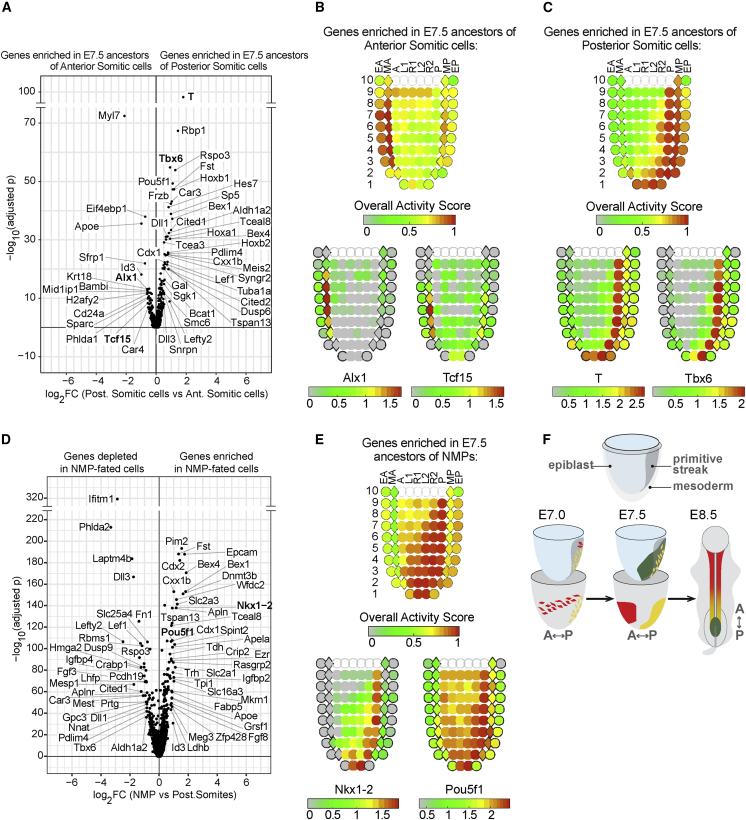
Anterior-Posterior Patterning of Paraxial Mesoderm during Gastrulation (A) Differential expression analysis of E7.5 cells with predicted posterior somitic fate versus E7.5 cells with predicted anterior somitic fate. Genes queried individually in the eGastrulation tool are highlighted in bold. See (B) and (C). (B) Overall “activity score” of the genes significantly enriched in the anterior trajectory (top) for E7.5 spatial data (Peng et al., 2019) and expression levels in log_10_ (FPKM+1) for selected genes (bottom) highlighted in bold font in (A). Cornplots were generated using the eGastrulation tool, where the embryo is represented by anatomical sections featuring anterior-posterior and left-right axes for sections in distinct proximal-distal regions (10 being most proximal and 1 most distal; EA, anterior endoderm; MA, anterior mesoderm; A, anterior epiblast; L1, anterior left lateral; R1, anterior right lateral; L2, posterior left lateral; R2, posterior right lateral; P, posterior epiblast; MP, posterior mesoderm; EP, posterior endoderm). (C) Overall “activity score” of the genes significantly enriched in the posterior trajectory (top), and expression levels in log_10_ (FPKM+1) for selected genes (bottom) highlighted in bold font in (A). See also legend for (B). (D) Differential gene expression of E7.5 cells with predicted NMP fate versus E7.5 cells with predicted somitic fate. Adjusted p value is calculated for differential gene expression in the cells with predicted NMP fate compared with either the anterior or the posterior somitic-fated cells. Log_2_ fold-change is shown for posterior somitic mesoderm cells only. Genes queried individually in the eGastrulation tool are highlighted in bold. See (E). (E) Overall “activity score” of the genes significantly enriched in the NMP trajectory (top), and expression levels in log_10_ (FPKM+1) for selected genes (bottom) highlighted in bold in (D). See also legend for (B). (F) Schematic of anterior-posterior patterning of paraxial mesoderm during gastrulation. Tissues biased to the E8.5 anterior somites are in red, those biased to the E8.5 posterior somites are in yellow, and those biased to the E8.5 NMP pool are in green. A, anterior; P, posterior. See also Figure S2; Table S2. Differential Expression Analysis between Cells Biased toward Anterior and Posterior Somitic Tissues, Related to Figures 3 and S3, Table S3. Genes Spatially Enriched in Anterior and Posterior Mesoderm, Related to Figures 3 and S3, Table S4. Differential Expression Analysis between Cells Biasedd toward NMPs and Somitic Tissues, Related to Figure 3.

The anterior and posterior somitic trajectories share early (E6.5–E7.0) transcriptional changes associated with gastrulation, as well as upregulation of somitic genes at E8.0–E8.5 (Figure 2C), but also show divergent expression at intermediate time points (E7.25–E7.75). Differential gene-expression analysis at E7.5 showed earlier *Tcf15* expression in the anterior trajectory, consistent with a more advanced somitic maturation compared with the posterior trajectory (Figures 2C and 3A; Table S2). Higher levels of *T* in the posterior trajectory were matched with higher expression of canonical regulators of somitogenesis, including *Tbx6* and members of the Wnt, Notch, retinoic acid, Fgf, and Nodal/Tgfb/BMP signaling pathways. Of note, formation of the earliest anterior somites has been observed in embryos that lack key somitic regulators such as *T*, *Tbx6*, *Wnt3a*, and *Fgfr1a* (Takada et al., 1994; Xu et al., 1999). E7.5 cells on the anterior somitic trajectory instead showed strong upregulation of the transcriptional regulator *Id3* as well as the homeobox transcription factor *Alx1*. Oscillating genes of the somitogenesis clock and wave-front model also had an overall reduced expression along the trajectory leading to anterior somitic tissues compared with that of posterior somitic tissues (Figure S3A).

We next interrogated the dynamics of gene expression along the trajectory toward the anterior paraxial mesoderm (Figure S3B). The transcription factor *Hand1* and adhesion molecule *Pmp22* showed early peaks of expression, the frizzled related protein *Sfrp1* and homeobox transcription factor *Alx1* peaked at a midpoint, and homeobox transcription factors of the *Irx* and *Prrx* family peaked at the end of the trajectory. Many of the above candidate regulators have not previously been implicated in somite development, yet the anterior trajectory culminates with induction of the somite master regulators *Tcf15* and *Meox1*.

### Parallel Spatial and Transcriptional Divergence of Distinct Somitic Mesoderm Programs

Complementary laser-capture microdissection experiments, profiling contiguous segments of approximately 20 cells, have been performed at equivalent stages of mouse development (Peng et al., 2019), thus allowing us to interrogate the spatial expression of genes differentially expressed between the posterior and anterior trajectories (Figure 3A). The E7.5 anterior somitic signature shows the strongest positional enrichment in anterior mesoderm, while the posterior signature is enriched in the posterior mesoderm and in the posterior epiblast sections of the Peng et al. dataset (Figures 3B and 3C). We also performed a similar analysis, in the opposite direction, by extracting the genes enriched, respectively, in anterior and posterior mesoderm at E7.5 from the Peng et al. dataset (Table S3) and assessing their expression in our single-cell atlas, which highlighted the expected populations of anterior and posterior somitic trajectories (Figure S3C; STAR Methods). This complementary analysis also highlighted additional expression sites (such as in lateral-plate mesoderm lineages) for genes on the anterior trajectory. Taken together, this supports the notion that at E7.5, posterior paraxial mesoderm precursor cells are still located close to the primitive streak, while the precursors of anterior paraxial mesoderm have already migrated to the anterior end of the embryo.

The clear separation of the two trajectories at E7.5 suggested they may be spatially segregated at earlier stages. We, therefore, employed a similar strategy to compare the two trajectories at E7.0 (Figure S3D; Table S2). Genes enriched in the E7.0 posterior paraxial mesoderm ancestors are most strongly associated with the primitive streak region, while genes specific to the anterior paraxial mesoderm ancestors show the highest enrichment in the mesoderm layer, suggesting that these cells have already ingressed through the primitive streak (Figure S3E). Interestingly, genes enriched in the anterior somitic trajectory are expressed in more proximal regions of the egg cylinder compared with those of the posterior trajectory, highlighting an additional spatial segregation of the two sets of ancestors.

Next, we characterized the trajectory leading to NMPs. Comparison with the somitic trajectories not only suggested an early divergence but also that ancestors of the posterior somitic tissues had a higher likelihood of contributing to the NMP trajectory than ancestors of anterior somitic tissues (Figures 2A and S3F). Differential gene-expression analysis between NMP and somitic trajectory cells at E7.5 showed higher levels of NMP-signature genes in NMP-fated cells (e.g., *Cdx1*, *Cdx2*, *Nkx1-2*, *Fst*, and *Grsf1*) (Gouti et al., 2017) and a higher expression of the epiblast markers *Dnmt3b*, *Epcam*, and *Pou5f1* (Figure 3D; Table S4). Conversely, NMP-fated cells had lower levels of the mesodermal genes *Mesp1*, *Aldh1a2*, *Cited1*, and *Rspo3* relative to the E7.5 ancestors of somitic tissues. This suggests that at E7.5, NMP ancestors have a more immature, epiblast-like signature compared with the early somite precursors. Consistently, spatial visualization of this NMP-enriched signature showed the highest scores in epiblast sections of the E7.5 embryo (Figure 3E).

This spatiotemporal transcriptional analysis supports a model whereby rostrocaudal patterning of the first somites is concomitant with gastrulation. Mesoderm cells biased to an anterior paraxial fate ingress earlier through the primitive streak and likely acquire their somitic identity anteriorly (marked by an upregulation of both *Tcf15* and *Meox1* after spatial segregation at E7.5), while cells destined for a more posterior paraxial fate undergo gastrulation later and develop posteriorly in the embryo. Finally, NMP ancestors remain in the posterior epiblast, where they acquire an NMP signature, sustained up until at least E8.5, the last time point sampled in the current atlas (Figure 3F).

### *T*^−/−^ Chimera Single-Cell Transcriptional Analysis Reveals Alterations in Common and Rare Cell Types

Given the role of *Brachyury* (*T*) in axial elongation, we were intrigued to observe that *T* was the most differentially expressed gene between the two early somitic trajectories (Figures 3A and S3D). The homozygous *T* mutant mouse model is embryonic lethal, with a severe arrest of axis elongation, notochord and allantois defects, and a kinked neural tube (Beddington et al., 1992; Chesley, 1935; Rashbass et al., 1991). To study the cell-autonomous effects of T knockout, we performed scRNA-seq on chimeric mouse embryos. We targeted *T* in a mouse embryonic stem cell line constitutively expressing tdTomato (Pijuan-Sala et al., 2019; see STAR Methods). We confirmed the disruption of *T* by sequencing the CRISPR-Cas9-targeted locus, which showed frameshift mutations and early stop codons precluding the generation of a functional protein, in two different clones (Figures S4A and S4B). Chimeric embryos generated with these two independent *T*^−/−^ clones were harvested at E8.5, mutant and wild-type (WT) cells sorted based on tdTomato fluorescence, and scRNA-seq performed on four independent pools of embryos, with 14,048 *T*^−/−^ and 13,724 WT single-cell transcriptomes passing quality control (Figure S4C; STAR Methods). Cell types were determined by mapping the chimeric embryo cells onto the reference atlas. As expected, mutant cells still expressed the *T* transcript, although at reduced levels (Figure S4D, in agreement with self-regulation of this transcription factor; Beisaw et al., 2018). Importantly and in line with mutation analysis by sequencing, there is no detectable T protein in *T*^−/−^ cells of chimeras (Figure S4E), likely due to severe effects on the stability and/or conformation of any residual peptide produced, as only the first 23% of the amino-acid sequence may be retained (Figure S4B).

**Figure 4:**
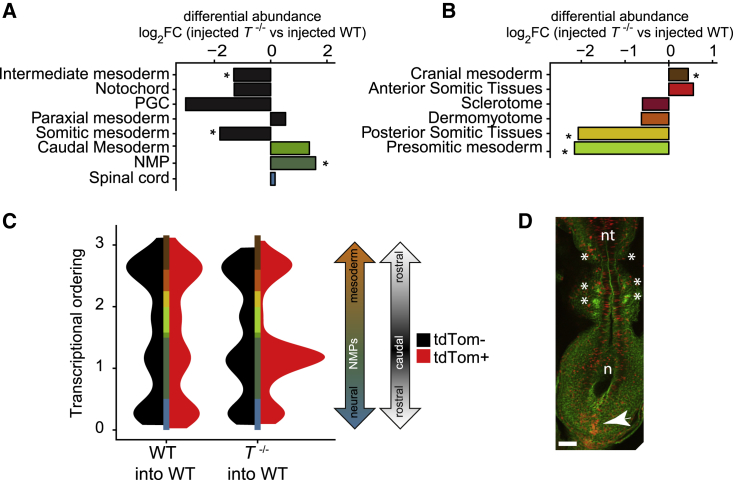
Development of *T*^−/−^ Cells in Chimeric Embryos Reveals a Differential Requirement of T in Two Developmental Trajectories Leading to Somitic Tissues (A) Differential abundance testing of cell types with most pronounced effects in *T*^−/−^ chimeras compared with WT controls, as well as other cell types relevant to axial elongation. ^∗^ BH-corrected p < 0.1, n = 4 independent experiments. (B) Differential abundance testing of somitic subclusters identified in Figure 1 in *T*^−/−^ chimeras compared with WT controls. ^∗^ BH-corrected p < 0.1, n = 4 independent experiments. (C) Density of mapped chimera cells along the one-dimensional diffusion pseudotime ordering from Figure 1. (D) Confocal image of a *T*^−/−^ chimeric embryo stained with phalloidin-alexa488 (green). Arrowhead points to accumulation of tdTom^+^ cells in the caudal region of the embryo (red). ^∗^ somites; nt, neural tube; N, node; Scale bar: 100 μm. See also Figure S4.

We performed differential abundance testing of cell types with reference to matched wild-type chimeras (Figure S4F; STAR Methods). This showed significantly reduced *T*^−/−^ cell contribution to intermediate and somitic mesoderm and increased contribution to NMPs (Figures 4A, S4F, and S4G). Other T-expressing tissues, e.g. notochord and primordial germ cells (PGCs), also showed changes in differential abundance, but below statistical significance, likely due to the low numbers of these cells at this time point. Interestingly, notochord cells showed perturbed gene-expression patterns in *T*^−/−^ cells (Figure S4H). Reduced contribution of *T*^−/−^ cells to the PGC lineage has been reported, but no quantitative analysis was performed (Aramaki et al., 2013). Given a quantitative reduction rather than total absence seen by scRNA-seq analysis of chimeric embryos, we quantified the numbers of presumptive PGCs present from the E7.5 (neural plate) to E8.5 (10 somite) stages in T-expressing embryos and then compared presumptive PGC numbers at E7.75 (headfold stage) with *T*^−/−^ embryos (Figures S4I and S4J). Counting presumptive PGCs in multiple embryos demonstrated that there is indeed a statistically significant reduction in the *T*^−/−^ samples. Thus, even in rare cell types such as notochord and PGCs, combining mouse chimeras with scRNA-seq reveals cell-autonomous roles for T.

### *T*^−/−^ Chimera Analysis Validates the Two Early Somitic Trajectories

We next focused on the subclusters of paraxial mesoderm defined in Figure 1. In line with previous findings (Beddington et al., 1992; Rashbass et al., 1991; Wilson et al., 1995), we observed a marked decrease in contribution to posterior somitic tissue and presomitic mesoderm, while cranial mesoderm and anterior somitic tissues showed only small changes in abundance (Figure 4B). This not only supports an essential cell-autonomous role for T specifically in the development of the E8.5 posterior somitic tissue but also confirms that the two sets of somites present at E8.5 emerge in molecularly distinct developmental events, as suggested by the two different trajectories defined above. To obtain a finer resolution, we assessed the distribution of chimeric cells mapped onto the transcriptional ordering from Figure 1C (STAR Methods). In WT chimeras, tdTomato^+^ (tdTom^+^) and tdTomato^-^ (tdTom^-^) cells were similarly distributed (p = 0.14, Kolmogorov-Smirnov [KS] test). By contrast, *T*^−/−^ cells accumulated in the caudal-most portion of the ordering in *T*^−/−^ chimeras (p ≤ 10^−15^, KS test; Figure 4C).

Since the mapping above was based on transcriptomic features, we next used confocal imaging to visualize the distribution of tdTom^+^ cells in chimeric embryos, confirming a caudal accumulation as predicted from scRNA-seq data (Figure 4D). Further examination of confocal Z-stacks of the primitive streak region suggested that caudal accumulation is primarily ectodermal and is, therefore, a consequence of failure to ingress through the primitive streak (Figure S4K), in agreement with prior observations (Wilson et al., 1995). Importantly, the confocal data also confirm that *T*^−/−^ cells contribute normally to anterior somitic tissues and to other tissues, namely cranial regions, endoderm, cardiac cells, allantois and extraembryonic mesoderm (Figures S4L and S4M). Of note, overrepresentation of *T*^−/−^ cells in the caudal NMP subset supports a previously proposed model, whereby higher levels of T favor ingression through the streak, while lower or absent T expression maintains cells in the streak region where they may ultimately contribute to the tail bud NMP pool (Wilson and Beddington, 1997).

### Characterization of NMP Over-Production in the Absence of T

**Figure 5:**
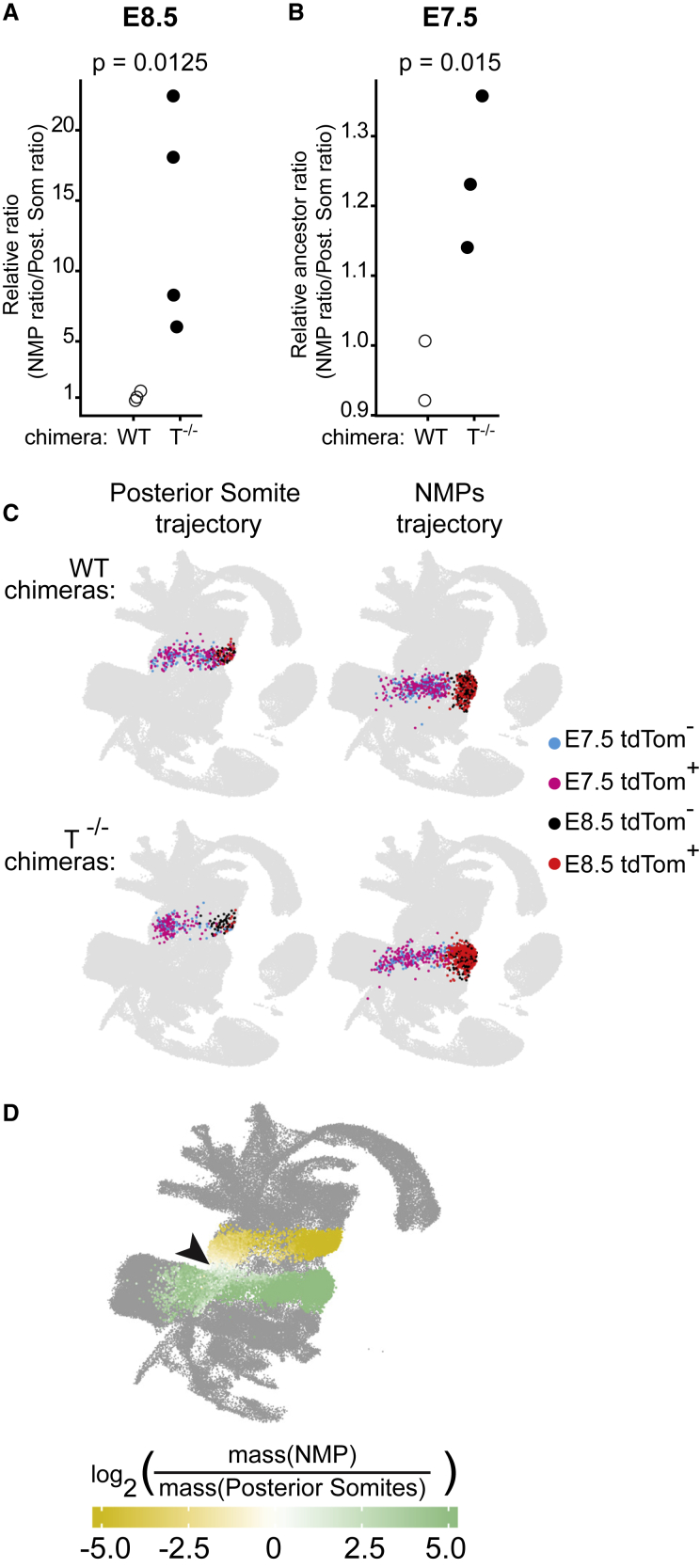
Assessing Allocation of *T*^−/−^ Cells to the NMP Pool (A) Relative contribution of injected cells to NMPs versus posterior somites in E8.5 chimeras (p values calculated by permutation). Each point is an independent experiment (pool of chimeric embryos) and calculated as: relative ratio = (number of tdTom^+^ NMPs / number of tdTom^−^ NMPs) / (number of tdTom^+^ posterior somite cells / number of tdTom^−^ posterior somite cells). Hollow circles, values for WT chimera assays; filled circles, values for *T*^−/−^ chimeras. (B) Relative contribution of injected cells to trajectories toward NMPs versus posterior somites in E7.5 chimeras, showing significant bias toward the NMP fate in *T*^−/−^ chimeras compared with WT (p values estimated by permutation; values plotted as in (A). (C) UMAP layout from Pijuan-Sala et al. (2019), highlighting mapped nearest neighbors of injected (tdTom^+^) and host cells (tdTom^−^) in E7.5 and E8.5 chimeras. (D) UMAP layout from Pijuan-Sala et al. (2019) with cells colored by their relative mass from NMP versus posterior trajectories. Values are capped at −5 and +5 for better legibility. Arrowhead highlights the nascent mesoderm cell subset with balanced mass (i.e., equal likelihood) for both trajectories, according to WOT.

To further investigate the relationships between the developmental trajectories for posterior somites and NMPs, we quantified the contribution of *T*^−/−^ cells across all replicates of our E8.5 chimeras to posterior somites and NMPs. To control for any potential differences in contribution to lineages intrinsic to the chimera assay, we considered the ratio of cell numbers in the injected population divided by the cell numbers in the host population for each lineage, in chimeras generated by injection of *T*^−/−^ and WT cells, respectively. This confirmed the change in balance between the two lineages (Figure 5A). We next asked whether cells lacking T might already be differentially abundant between these two trajectories at E7.5 (Figure 2B). We, thus, generated a new set of chimeras that were harvested at E7.5 and analyzed by scRNA-seq (STAR Methods; Figures 5B and S5A–S5C). Quantitative analysis across replicate experiments confirmed the trend toward a reduced contribution to the posterior somitic trajectory, although, at this stage, mutant cells were only slightly overrepresented in the NMP trajectory (Figure 5B). Two other observations are noteworthy. For the E8.5 chimeras, there is still a small contribution of *T*^−/−^ cells to posterior somitic mesoderm, meaning that the *T*^−/−^ phenotype is not fully penetrant at this stage (Figure S4G). Second, when the E7.5 chimera cells are mapped onto the landscape, a minority of cells is fairly far advanced, whereas the bulk still sits in a territory that overlaps with the NMP trajectory (Figures 5C and 5D). A model, therefore, emerges where the earlier cells contributing to the posterior somites may do so even in the absence of T, whereas the rest may be diverted along the NMP trajectory.

### *T*^−/−^ NMPs Do Not Show Molecular Evidence of an Early Fate Switch

**Figure 6:**
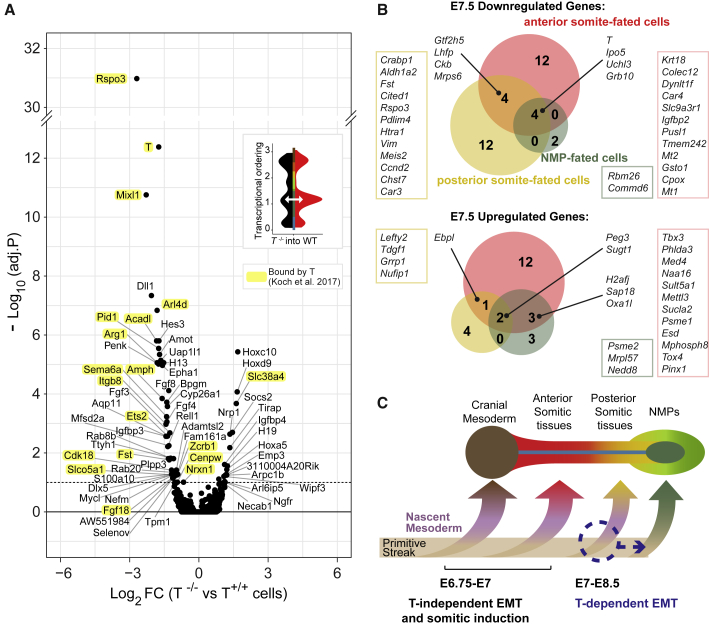
Role of T in the Formation of the First Posterior Somites and Establishment of the NMP Pool (A) Differential gene expression between E8.5 mutant cells accumulated in an NMP state and their WT counterparts within chimeras (see inset and Figure 4C). Genes previously found to be bound by T (Koch et al., 2017) are highlighted in yellow. (B) Differentially expressed genes in tdTom^+^*T*^−/−^ cells in E7.5 chimeric embryos compared with their tdTom^−^ WT counterparts (adjusted p < 0.1), within the transcriptomes mapping to each of the developmental trajectories highlighted in Figures 2A and 2B. Genes also identified as differentially expressed in control chimeras (injected with WT tdTom^+^ cells) or significantly correlated with the tdTomato transcript were considered as results of a chimera assay-related technical bias and excluded from the analysis (see Figures S6D–S6F). (C) Working model for cell-autonomous role of T in the formation of the first somites during gastrulation. See also Figure S6; Tables S5 and S6.

The accumulation of *T*^−/−^ cells in an NMP transcriptional state in E8.5 chimeras prompted us to characterize this overrepresented mutant NMP subset by differential gene-expression analysis (Figures 6A and S6A). The majority (75%) of genes differentially expressed in the absence of T were downregulated, suggesting that, in these cells, T functions mostly as a transcriptional activator (Figure 6A; Table S5). Moreover, 18 of the significantly downregulated genes have previously been identified by ChIP-seq as direct targets of T in *in vitro* NMP models (Koch et al., 2017), which is significantly more than expected by chance (18 of 47 genes; p < 10^−11^, Fisher’s Exact test; Figure 6A, genes highlighted in yellow).

Genes downregulated in *T*^−/−^ NMPs include major elements of the canonical somitogenesis signaling pathways: Wnt (*Rspo3*), Fgf (*Fgf3*, *Fgf4*, *Fgf8*, and *Fgf18*), Notch (*Dll1* and *Hes3*), and retinoic acid (*Cyp26a1*). This is consistent with the previously reported positive feedback loops between T and these pathways during axial extension (Diez del Corral et al., 2003; Hubaud and Pourquié, 2014; Kumar and Duester, 2014; Vermot and Pourquié, 2005). Less well implicated but likely also important regulators include the cell-cell adhesion and signal transduction genes *Sema6a*, *Epha1*, *Itgb8*, *Igfbp3*, *Penk*, *Nrxn1*, and *Fst*. The transcription factors *Mixl1*, *Ets2*, *Mycl*, and *Dlx5* were also downregulated and may, therefore, play previously unsuspected roles in NMP regulation and somitogenesis downstream of T.

It was proposed that the multipotent nature of NMPs relies on cross-antagonism between *T* and the neural-determining factor, *Sox2*, where each serves as a lineage-determining factor (Gouti et al., 2017; Koch et al., 2017). Furthermore, in our analysis of gene-expression dynamics along the NMP trajectory, we observed a decline in *T* transcript concurrent with the increase in *Sox2* between E7.5 and E8.5 (Figure 2C), which would support this model. Accordingly, *T*^−/−^ NMPs would be expected to express higher levels of *Sox2* than WT NMPs, which would, in turn, increase the production of spinal cord progenitors (Takemoto et al., 2011). However, neither *Sox2*, nor a broader neural signature, were upregulated in *T*^−/−^ NMPs (Figures 6A, S6B, and S6C). Moreover, spinal cord cells were not overproduced in the *T*^−/−^ chimeras (Figure 4A). Our analysis of primary cells, therefore, argues against a cell-autonomous mutually repressive model of *T* and *Sox2* as early NMP fate determinants.

To investigate earlier molecular consequences of *T* knockout, we next performed differential gene-expression analysis within E7.5 chimeras, focusing on the tdTom^+^ and tdTom^−^ cells mapping to each trajectory (Figures 6B and S6D–S6F; Table S6). There was little overlap between the sets of deregulated genes across the different trajectories, consistent with trajectory-specific effects at this early time point. Among the genes upregulated in cells biased to anterior somitic tissues was the T-box family transcription factor *Tbx3*. Cells biased to posterior somitic tissues showed downregulation of genes involved in cell migration including *Vim*, *Pdlim4*, and *Htra1* (Fu et al., 2019; Singh et al., 2014; Ye and Weinberg, 2015). These cells also displayed downregulation of *Cited1*, previously shown to label specifically cells that have ingressed through the primitive streak (Garriock et al., 2015). Of note, genes related to an incomplete EMT state (Figures S2B–S2D) were not affected in *T*^−/−^ NMP ancestors at any of the analyzed time points (Figures 6A and 6B).

Taken together, these results suggest that the precursors of anterior mesoderm are capable of undergoing gastrulation in the absence of T. Precursors of more posterior somites reach the streak later in development and require T to activate genes involved in EMT. In the absence of T, they remain in the streak region, where they may contribute to the developing pool of NMPs (Figure 6C).

## Discussion

By integrating computational methods with scRNA-seq of embryonic chimeras, we inferred three distinct trajectories from pluripotent epiblast cells toward somite development. We revealed previously unknown dynamic gene expression during the emergence of the anterior-most somites, accompanied by a clear spatial separation at E7.5. Analysis of *T*^−/−^ chimeras validated these trajectories, suggested reallocation of early posterior somite progenitors to the NMP pool in the absence of T, and supported a model whereby T does not inhibit expression of *Sox2* in NMPs.

To infer developmental trajectories, we applied WOT (Schiebinger et al., 2019), which has the key advantage, compared with many trajectory inference methods, of incorporating real-time information when analysing time-course datasets. Methods that do not take real-time information into account can produce erroneous assignments when similar cell types emerge over an extended period of time or in “waves”. WOT allowed us to disentangle transcriptional trajectories with relatively similar signatures (in relation to the whole embryonic landscape), but with different times of developmental emergence. Importantly, additional independent analyses using spatial transcriptomic data, as well as the distinct effects of the *T* knockout in the -chimera assays were consistent with the trajectories inferred from the scRNA-seq data.

Our results support a model whereby the first somites develop from precursors that ingress early through the primitive streak and migrate anteriorly, concurrently with the precursors of other anterior mesoderm tissues. This agrees with previous fate-mapping experiments where precursors of the first pairs of somites are found in the same regions of the primitive streak as cardiac and cranial mesoderm, ingressing at around E7.0 (Kinder et al., 1999). The anterior somitic trajectory was characterized by higher levels of previously identified marker genes of lateral-plate mesoderm (e.g., *Hand1*, *Prrx1*, and *Prrx2*), also suggesting this shared ontogeny. Different timing of ingression is further supported by higher expression levels of caudal Cdx/Hox transcription factors in the E8.5 posterior paraxial tissues compared with anterior paraxial tissues, reflecting a later timing of ingression of precursors of posterior paraxial mesoderm (Forlani et al., 2003). One of the most noteworthy observations here is molecular convergence, where both the early anterior and posterior trajectories ultimately acquire a paraxial transcriptional identity, yet through journeys that are temporally, spatially, and molecularly distinct.

Analysis of *T*^−/−^ embryos indicated that the anterior somitic tissues identified here correspond to the first somite subsets, previously shown to form in the absence of T (Chesley, 1935). In E7.5 chimeric embryos, genes involved in cell migration were specifically downregulated in posterior somite-fated *T*^−/−^ cells, providing a molecular explanation for previous reports where impaired cell migration was suggested to cause the observed accumulation of mutant cells in the remnants of the primitive streak of chimeric embryos (Wilson and Beddington, 1997; Wilson et al., 1995). Our data further show that E8.5 caudal accumulation of *T*^−/−^ cells is coupled with the acquisition of an aberrant NMP signature, consistent with the model proposed by Wilson and Beddington (1997), where primitive streak cells harboring lower levels of T protein remain in the streak throughout gastrulation and contribute to the NMP pool of the developing tail bud to fuel subsequent axial elongation. Further studies will be required to functionally validate whether different levels of T regulate the allocation of individual streak cells to paraxial mesoderm or NMPs in the wild-type setting.

The ability of anterior paraxial mesoderm precursors to ingress through the streak and migrate anteriorly in the absence of T suggests they rely on other factors. Other members of the T-box protein family may play this role: the anterior somite-fated cells ingress through the streak before E7.0, within the window of *Eomes* expression during gastrulation (Figure 2C) and with considerable overlap with T in gene targets (Tosic et al., 2019). Our analysis revealed *Tbx3* as another possible candidate, with specific upregulation at the start of the developmental trajectory toward anterior somitic tissues, and in the E7.5 *T*^−/−^ cells fated to the anterior somitic tissues (Figures 6B and S3B).

As in prior mouse and zebrafish studies, we observed a residual contribution of *T*^−/−^ cells to the posterior somitic tissues (Martin and Kimelman, 2010; Wilson and Beddington, 1997). While expression of somitic markers had not been tested in these studies, our results suggest that some of these residual cells are indeed correctly transcriptionally patterned as somitic mesoderm.

Characterization of *T*^−/−^ NMP-like cells suggested a model where T is required for NMPs to move down a somitic differentiation path, but where T has little bearing on NMPs moving along the neural lineage. The observation that many *T*^−/−^ NMPs become trapped in the primitive streak, rather than produce excess neural tissue, suggests that at the single-cell level in the intact embryo, many NMPs may not have both somitic and neural differentiation options available to them, possibly due to spatial constraints. Indeed, although *in vivo* lineage tracing suggest widespread bipotency for larger NMP clones (Tzouanacou et al., 2009), heterotopic transplantation and live-cell imaging studies suggest that many cells with NMP potential will only differentiate into one lineage in the embryo (Wood et al., 2019; Wymeersch et al., 2016).

In the present report, we show that single-cell transcriptional analysis of entire embryos provides a complementary approach toward a better understanding of long-standing questions in developmental biology. Moving forward, the ability to couple such unbiased transcriptional profiling with information about a cell’s location within the organism will further enable new biological discovery. Together with appropriate functional experiments, this promises to open an exciting new chapter in developmental biology, where hypotheses can be investigated *in vivo*, at single-cell resolution, genome-wide scale, and at the level of the whole organism.

## STAR★Methods

### Key Resources Table

**Table:** 

REAGENT or RESOURCE	SOURCE	IDENTIFIER
**Antibodies**		

Human/Mouse Brachyury Affinity Purified Polyclonal Ab antibody	R and D Systems	Cat# AF2085; RRID: AB_2200235
Donkey anti-Goat IgG (H+L) Cross-Adsorbed Secondary Antibody, Alexa Fluor 647	Thermo Fisher Scientific	Cat# A-21447; RRID: AB_2535864

**Chemicals, Peptides, and Recombinant Proteins**		

VECTASHIELD Mounting Medium	Vector Laboratories	Cat# H-1000; RRID: AB_2336789
Alexa Fluor 488 Phalloidin	Thermo Fisher Scientific	Cat# A12379

**Critical Commercial Assays**		

Chromium Single Cell 3′ Library & Gel Bead Kit v2	10X Genomics	PN-120237
Chromium Single Cell 3′ Chip Kit v2	10X Genomics	PN-120236

**Deposited Data**		

*T* chimera single-cell RNA-seq	This paper	ArrayExpress: E-MTAB-8811
New wild-type chimera single-cell RNA-seq	This paper	ArrayExpress: E-MTAB-8812
Existing wild-type chimera single-cell RNA-seq	Pijuan-Sala et al., 2019	ArrayExpress: E-MTAB-7324
Mouse gastrulation single-cell RNA-seq atlas	Pijuan-Sala et al., 2019	ArrayExpress: E-MTAB-6967

**Experimental Models: Cell Lines**		

tdTomato+ mESC line (male, karyotypically normal)	Pijuan-Sala et al. 2019	B6xtdTom+ cl12-F10
*T*^-/-^ tdTomato+ mESC Clone 1	This paper	B6xtdTom+ cl12-F10-B6
*T*^-/-^ tdTomato+ mESC Clone 2	This paper	B6xtdTom+ cl12-F10-C6

**Experimental Models: Organisms/Strains**		

Heterozygous T/+ BTBR/Pas mice	Rashbass et al., 1994	Heterozygous T/+ BTBR/Pas mice
C57BL/6 wild type mice	Charles River	C57BL/6J (JAX™ Mice Strain)

**Oligonucleotides**		

F-primer for embryo genotyping (Figures S4I and S4J) CCAGTTGACACCGGTTGTTACA	Sigma Aldrich	N/A
R-primer for embryo genotyping (Figures S4I and S4J) TATCCCAGTCTCTGGTCTGT	Sigma Aldrich	N/A
F-primer for embryo genotyping (Figures S4I and S4J; positive control) GCGCCAGTGCAGGGAAGATTGGAA	Sigma Aldrich	N/A
R-primer for embryo genotyping (Figures S4I and S4J; positive control) GATATGACTGGGCCAGACGGAAA	Sigma Aldrich	N/A
T locus targeting gRNA1: TGACGGCTGACAACCACCGC	Sigma Aldrich	N/A
T locus targeting gRNA2: GCCCCAAAATTGGGCGAGTC	Sigma Aldrich	N/A
F-primer for NGS of T-targeted mESC clones TCGTCGGCAGCGTCAGATGTGTATAA GAGACAGTCCCGGTGCTGAAGGTAAAT	Sigma Aldrich	N/A
R-primer for NGS of T-targeted mESC clones GTCTCGTGGGCTCGGAGATGTGTA TAAGAGACAGCCTGCTTAACCC TCATCAGC	Sigma Aldrich	N/A

**Recombinant DNA**		

pX458 plasmid	Addgene	#48138

**Software and Algorithms**		

Cellranger	Zheng et al., 2017	https://support.10xgenomics.com/single-cell-gene-expression/software/downloads/latest?
Scran	Lun et al., 2016	http://bioconductor.org/packages/release/bioc/html/scran.html
DropletUtils	Griffiths et al., 2018	http://bioconductor.org/packages/release/bioc/html/DropletUtils.html
Uwot	McInnes et al. 2020, https://arxiv.org/abs/1802.03426	https://cran.r-project.org/web/packages/uwot/index.html
Destiny	Angerer et al. 2016	http://bioconductor.org/packages/release/bioc/html/destiny.html
FIJI	Schindelin et al. 2012	https://imagej.net/Fiji

### Resource Availability

#### Lead Contact

Further information and requests for resources and reagents should be directed to and will be fulfilled by the Lead Contact, John C. Marioni (john.marioni@cruk.cam.ac.uk).

#### Materials Availability

Mouse embryonic stem cell lines generated in this study are available upon request.

#### Data and Code Availability

Raw sequencing data is available on Arrayexpress: T chimeras – E-MTAB-8811; WT chimeras – E-MTAB-7324 (as used in Pijuan-Sala et al., 2019) and E-MTAB-8812 (newly generated). Original data have been deposited with accession numbers: Arrayexpress: E-MTAB-8811, E-MTAB-8812. Processed data is available from the Bioconductor package MouseGastrulationData (https://bioconductor.org/packages/release/data/experiment/html/MouseGastrulationData.html). This includes the single-cell RNA-seq data directly, as well as the NMP orderings, and somitogenesis trajectory labels used in this manuscript. An online visualisation tool is available at https://marionilab.cruk.cam.ac.uk/EarlySomites2020/.

### Experimental Models and Subject Details

#### Cell Lines

All mouse embryonic stem cell lines were expanded under the 2i+LIF conditions (Ying et al., 2008), in a humidified incubator at 37°C and 7% CO_2_, and routinely tested negative for mycoplasma infection. A male, karyotypically normal, tdTomato-expressing mouse embryonic stem cell line was derived from E3.5 blastocysts obtained by crossing a male ROSA26tdTomato (Jax Labs – 007905) with a wildtype C57BL/6 female. Competence for chimera generation was assessed using morula aggregation assay. Targeting of the *T* locus was performed using the CRISPR/Cas9 system (see Method Details), mutant clones were assessed by next-generation sequencing (see Figure S4). Two mutant clones were used to generate *T*^-^^/-^ embryonic chimeras.

#### Mouse Models

All procedures were performed in strict accordance to the UK Home Office regulations for animal research. Chimaeric mouse embryos were generated under the project licence number PPL 70/8406. Animals used in this study were 6-10 week-old females, maintained on a lighting regime of 14 hours light and 10 hours darkness with food and water supplied ad libitum. For chimera generation, E3.5 blastocysts were derived from wildtype C57BL/6 matings, and after injection of the mutant cells, the resulting chimaeric embryos were transferred to C57BL/6 recipient females at 0.5 days of pseudopregnancy following mating with vasectomised males.

### Method Details

#### Somitic Trajectory Analysis from Atlas Data

##### Subclustering the Atlas Paraxial Cell Types

To dissect the Paraxial Mesoderm sub-populations present in the E8.5 embryo, cells from the reference Atlas (Pijuan-Sala et al., 2019) belonging to E8.5 time-point and to the cell types “Paraxial Mesoderm” and “Somitic Mesoderm” were extracted and re-clustered using igraph's Louvain algorithm. Clustering was performed on Mutual Nearest Neighbours (MNN) batch corrected principal components (top 50), and the resulting subclusters were annotated using differentially expressed genes.

##### Transcriptional Ordering of Axial Elongation Cell Types

The Atlas data were subset to E8.5 cells of spinal cord, NMP, caudal epiblast, caudal mesoderm, somitic mesoderm, and paraxial mesoderm cell types. A 50-dimensional principal component (PC) space was generated from these cells from log-transformed normalised gene counts (with an added pseudocount of 1), considering only highly-variable genes (HVGs, see Selection of HVGs in the “quantification and statistical analysis” section, below). Expression levels for each gene were centred, but not scaled, prior to PC computation. PCs were calculated using the irlba package. To ensure that the atlas manifold was continuous in the PC subspace, and so that batch-effects could not affect mapping of chimera data, it was batch-corrected as described below (see ‘Batch correction’). As the manifold is largely a one-dimensional structure (see Figure 1A), it was summarised into a one-dimensional ordering using diffusion pseudotime (DPT; Haghverdi et al., 2016). DPT was computed from a diffusion map, itself computed from the atlas cells in the PC subspace, with DPT ordering from the spinal cord cell with most extreme value of the first diffusion component.

##### Identifying Somitic Developmental Trajectories

To reconstruct the lineages of cells in the reference atlas, we used the W-OT package 1.0.7 (Schiebinger et al., 2019) to estimate the sequence of ancestor distributions at earlier time points. Cells were allocated to the trajectory of their largest endpoint mass contribution, or to multiple trajectories if their mass contribution was at least 90% as large as their largest endpoint mass contribution (to capture apparently uncommitted cells).

##### Spatial Domains of Trajectory-Specific Expression Signatures

Genes that defined the posterior and anterior somitic trajectories at E7.0 and E7.5 (determined by differential expression, with adjusted P value < 0.1; differential expression testing was performed using the scran function findMarkers using default parameters) were introduced into the Gene Activity Score tool provided by the eGastrulation database (http://egastrulation.sibcb.ac.cn/; Peng et al., 2019) to generate 2-dimensional “corn plots”. For the reverse analysis (Figure S3C), signature genes enriched in anterior and posterior mesoderm domains in the Peng et al. dataset were retrieved using the “Gene Search by Pattern” tool provided by the eGastrulation database. The following patterns were used as input: anterior – value of 80 for rows 3 to 7 of MA column (remaining slots were given 0); posterior – value 80 for rows 3 to 7 of MP column and value 60 for rows 3 to 7 of P column. Cutoff for correlation analysis: RCC > 0.4. We transformed the atlas expression levels onto a common scale (as a Z-score for each gene), and plotted the average Z-score of the Peng et al. signature genes on our transcriptional Atlas layout, which highlighted the expected populations of anterior and posterior somitic trajectories (Figure S3C). For details on gene expression comparisons along trajectories, see “quantification and statistical analysis” section below.

#### Chimera Generation and Sequencing

##### Embryo Collection

All procedures were performed in strict accordance to the UK Home Office regulations for animal research under the project license number PPL 70/8406.

##### Chimera Generation

TdTomato-expressing mouse embryonic stem cells (ESC) were derived as previously described (Pijuan-Sala et al., 2019). Briefly, ESC lines were derived from E3.5 blastocysts obtained by crossing a male ROSA26tdTomato (Jax Labs – 007905) with a wildtype C57BL/6 female, expanded under the 2i+LIF conditions (Ying et al., 2008) and transiently transfected with a Cre-IRES-GFP plasmid (Wray et al., 2011) using Lipofectamine 3000 Transfection Reagent (ThermoFisher Scientific, #L3000008) according to manufacturer’s instructions. A tdTomato-positive, male, karyotypically normal line, competent for chimera generation as assessed using morula aggregation assay, was selected for targeting T. Two guides were designed using the http://crispr.mit.edu tool (guide 1: TGACGGCTGACAACCACCGC; guide 2: GCCCCAAAATTGGGCGAGTC) and were cloned into the pX458 plasmid (Addgene, #48138) as previously described (Ran et al., 2013). The obtained plasmids were then used to transfect the cells and single transfected clones were expanded and assessed for Cas9-induced mutations. Genomic DNA was isolated by incubating cell pellets in 0.1 mg/ml of Proteinase K (Sigma, #03115828001) in TE buffer at 50°C for 2 hours, followed by 5 min at 99°C. The sequence flanking the guide-targeted sites was amplified from the genomic DNA by polymerase chain reaction (PCR) in a Biometra T3000 Thermocycler (30 sec at 98°C; 30 cycles of 10 sec at 98°C, 20 sec at 58°C, 20 sec at 72°C; and elongation for 7 min at 72°C) using the Phusion High-Fidelity DNA Polymerase (NEB, #M0530S) according to the manufacturer’s instructions. Primers including Nextera overhangs were used (F- TCGTCGGCAGCGTCAGATGTGTATAAGAGACAGTCCCGGTGCTGAAGGTAAAT; R- GTCTCGTGGGCTCGGAGATGTGTATAAGAGACAGCCTGCTTAACCCTCATCAGC), allowing library preparation with the Nextera XT Kit (Illumina, #15052163), and sequencing was performed using the Illumina MiSeq system according to manufacturer’s instructions. Two ESC clones showing frameshift mutations in exon 2 resulting in the functional inactivation of T were selected for injection into C57BL/6 E3.5 blastocysts. A total of 17 chimaeric embryos were harvested at E8.5, dissected, and single-cell suspensions were generated from three independent pools of embryos by TrypLE Express dissociation reagent (Thermo Fisher Scientific) incubation for 7-10 minutes at 37°C under agitation. Single-cell suspensions were sorted into tdTom^+^ and tdTom^-^ samples using a BD Influx sorter with DAPI at 1μg/ml (Sigma) as a viability stain for subsequent 10X scRNA-seq library preparation (version 2 chemistry), and sequencing using the Illumina HiSeq 4000 platform, which resulted in 13,724 tdTom- and 14,048 tdTom^+^ cells that passed quality control (see “Single-cell RNA sequencing analysis” below). To exclude transcriptional effects intrinsic to the chimera assay, chimaeric embryos were generated by injecting the parental tdTom^+^ *T*^+/+^ (WT) line into C57BL/6 E3.5 blastocysts and processed as for the *T*^−/-^ samples. Three independent embryo pools with a total of 13 embryos were used for scRNA-seq, and 1,077 tdTom- and 2,454 tdTom^+^ cells passed quality control.

##### Embryo Staining and Imaging

Following dissection, embryos were washed in PBS and fixed in 4% paraformaldehyde (PFA, Thermo Scientific) for 1 hour at room temperature. They were then washed three times for 15 minutes in wash buffer (0.1% fraction 5 bovine serum albumin, 0.1% Tween20, 5% DMSO, 0.1% Triton-X in PBS), permeabilized overnight at 4°C in permeabilization buffer (0.1% fraction 5 bovine serum albumin, 0.1% Tween20, 5% DMSO, 0.25% Triton-X in PBS) and washed three times for 15 minutes in wash buffer. Embryos were then incubated overnight in blocking solution (5% donkey serum and 1% BSA in wash buffer) at 4°C, washed three times for 15 minutes in wash buffer and incubated overnight at 4°C in blocking solution containing the goat anti mouse Brachyury primary antibody (1:200, R&D Systems, cat# AF2085). After three 15 minute washes, Phalloidin-AlexaFluor488 (Thermofisher Scientific) was added 1:1000 and 4′,6-Diamidino-2-phenylindole dihydrochloride (DAPI, Sigma) was added at 200ng/ml with the donkey anti-goat Alexa647 antibody (1:500, Invitrogen, cat# A21447) in blocking solution for another overnight incubation at 4°C. Embryos were then washed three times for 15 minutes in wash buffer and mounted in Vectashield mounting media (Vector laboratories, cat# H-1000) and imaged in a Confocal Leica TCS SP5 microscope. Images were captures with the Leica Application Suite software and processed for publication using Fiji.

#### Quantification of Primordial Germ Cells

Following dissection, embryos were stained for Alkaline phosphatase activity as described previously (Ginsburg et al., 1990). Briefly, embryos were fixed in absolute ethanol with 12.5% glacial acetic acid at 4°C for 1 hour, followed by two 24h incubations in absolute ethanol at 4°C and two 1h washes in chloroform. They were mounted in wax, sectioned and incubated in freshly made staining solution (0.1mg/ml 1-Naphthyl phosphate, 0.5% borax solution, 0.5mg/ml Fast Red TR salt and 0.6% MgCl2, pH 9.2) for 15-30 minutes. For genotyping, extra-embryonic tissues of each embryo were digested with Proteinase K and tested by polymerase chain reaction for the presence of a 310bp region including the 3’ coding region of the T gene, missing in *T*^-/-^ embryos (primers: CCAGTTGACACCGGTTGTTACA and TATCCCAGTCTCTGGTCTGT). A 350bp fragment spanning the homeodomain of Hox 2.1 was used as a positive control (primers: GCGCCAGTGCAGGGAAGATTGGAA and GATATGACTGGGCCAGACGGAAA) (Rashbass et al., 1994).

#### Single-Cell RNA Sequencing Analysis

##### 10X Data Pre-processing

Raw files were processed with CellRanger 2.1.1 using default mapping arguments, with reads mapped to the mm10 genome and counted with GRCm38.92 annotation, including tdTomato sequence. This older annotation was used to ensure consistency with the reference atlas (Pijuan-Sala et al., 2019). Processed data and raw count matrices are available in the Bioconductor package MouseGastrulationData.

##### Swapped Molecule Removal

Molecule counts that derived from barcode swapping were removed from all 10X samples by applying the DropletUtils function swappedDrops (default parameters) to groups of samples (where a sample is a single lane of a 10X Chromium chip) that were multiplexed for sequencing.

##### Cell Calling

Cell barcodes that were associated with real cell transcriptomes were identified using emptyDrops (Lun et al., 2019), which assesses whether the RNA content associated with a cell barcode is statistically significantly distinct from the ambient background RNA present within each sample. A minimum UMI threshold was set at 5,000, and cells with an adjusted p-value < 0.01 (BH-corrected) were considered for further analysis.

##### Quality Control

Cells with mitochondrial gene expression fractions greater than 2.52% and 2.90%, for the *T*^-/-^ chimeras and WT chimeras respectively, were excluded. These thresholds were determined by the data – we considered a median-centred MAD-variance normal distribution; cells with mitochondrial read fraction “outside” of the upper end of this distribution were excluded (adjusted p-value < 0.05; BH-corrected).

##### Normalisation

Transcriptome size factors were calculated for each dataset separately (*T*^-/-^ chimeras, WT chimeras), using computeSumFactors from the R scran package (Lun et al., 2019), using default parameters. Raw counts for each cell were divided by their size factors, and the resulting normalised counts were used for further processing.

#### Visualisation of Single-cell RNA Sequencing Data

##### Batch Correction

Batch-effects were removed using the fastMNN function in scran on the first 50 PCs, computed from the HVG-subset logcount matrix. Default parameters were used. When correcting the reference atlas (Pijuan-Sala et al., 2019), correction was performed first between the samples within each time-point, merging sequentially from the samples containing the most cells to the samples containing the least. Time-points were then merged from oldest to youngest. When correcting the chimeras, correction was performed on all samples within a genotype first, from largest sample to smallest, then across the two genotypes. ***UMAPs*** were calculated using the uwot R package with default parameters except for min_dist = 0.7. ***Diffusion maps*** were calculated using the R package destiny, with function DiffusionMap, using default settings. Batch-corrected principal components were used.

#### Chimera Cell Type Annotation

To annotate the cell types in the chimaeric embryos, we performed a transcriptional mapping to a large reference atlas of mouse embryonic development (Pijuan-Sala et al., 2019). Each stage of the atlas was sub-sampled at random to 10,000 cell libraries (i.e., including the technical artefacts of doublets and stripped nuclei) at each time-point. Cells from the mixed time-point were excluded. This subsampling reduces potential bias due to the different number of cells captured at each stage. Stages E6.5 and E6.75 contained fewer cells than other stages (3,697 and 2,169 respectively) and were not downsampled; however we do not expect cells from E8.5 or E7.5 chimeras to map to these time-points. A shared 50-dimensional PC subspace was constructed from the subsampled cells from the atlas, and all chimera cells that were to be mapped. Batch-correction was then performed on the atlas cells in the PC space, as described above (Batch correction), to construct a single contiguous reference manifold. Samples to be mapped were then independently mapped onto the newly-corrected atlas data (scran function fastMNN), and the 10 nearest cells (by Euclidean distance) in the atlas to each chimera cell were recorded. Mapped time-point and cell type of chimera cells were defined as the most frequent of those of its 10 nearest-neighbours. Ties were broken by choosing the stage or cell type of the cell that had the lowest distance to the chimera cell. Cells that mapped to doublet- or stripped nucleus-labelled cells were excluded from downstream analyses. For cell type differential abundance testing in chimaeric embryos, see “quantification and statistical analysis” section below.

#### Mapping Chimera Cells onto the Atlas Backbone

To map chimera cells onto their appropriate positions on the atlas manifold, they were mapped onto it using a strategy similar to that used in Batch correction (above). Individual samples (i.e. one 10X channel) of the E8.5 chimera datasets were mapped onto the corrected atlas using fastMNN, using coordinates from the PC subspace. This operation was repeated for each chimera sample, retaining the mapped coordinate values for each cell. Performing this operation in parallel across samples prevents any mapped chimera cells affecting the future mapping of other samples. For the spinal cord to head mesoderm ordering, mapping was performed only using cells from the relevant cell types. DPT values (i.e., ordering positions) were inferred for chimera cells by considering the mean DPT value for the 5 nearest atlas cells in the PC space, after performing the per-sample mapping. This value of DPT is, effectively, the position of a chimera cell along the atlas backbone. For mapping chimera cells to somite trajectories through the atlas, chimera cells were mapped to the whole atlas (excluding cells from the “mixed gastrulation” atlas time-point, and with the subsampling described above), as above for cell type labelling. As for the previous approach, chimera cells were considered a part of a trajectory if the most common trajectory state of their 10 nearest neighbours was one of the somite trajectories. For differential gene expression analyses, see “quantification and statistical analysis” section below.

### Quantification and Statistical Analysis

#### Analysis of Single-Cell Datasets

##### Selection of HVGs

HVGs were calculated using trendVar and decomposeVar from the scran R package, with loess span 0.05. Genes that had significantly higher variance than the fitted trend (BH-corrected p < 0.05) were retained. Genes with mean log_2_(normalised count) < 10^-3^, genes on the Y chromosome, *Xist*, and *tdTomato* were excluded.

##### Gene Expression Comparisons along Trajectories

First, we selected genes that were variable along any of the three trajectories. We took the union of the genes calculated in each of the three trajectories, calculated according to the following procedure, considering only the cells from that trajectory: HVGs were first identified (see Selection of HVGs, below), and their mean expression level at each time-point was calculated; an order three polynomial linear model fit was compared to an intercept-only model by F-test (i.e., R function *anova*). We considered genes to be variable along a trajectory if the polynomial fit was significantly better than the intercept-only model (BH-corrected p < 0.1). In a pairwise manner across the three trajectories, we then tested these genes for differences in expression along them. As above, we calculated the mean expression level in each trajectory for the genes at each time-point. We then fitted a null model of an order three polynomial (i.e., the same model as for selecting genes above, except with the model using data from two, rather than one, trajectories at each time point). The alternative model allowed for trajectory-specific coefficients for each coefficient of the order three polynomial. We then compared the fit of the two models (by F-test) and considered genes to show different patterns of expression along the trajectories if they were fit better by the alternative model (BH-corrected FDR < 0.01). If the latter model fits better than the null, this suggests that the data are better described by different polynomials for each trajectory.

#### Overlap Computation (GSEA)

Following pair-wise comparisons of expression dynamics along the entire length of transcriptional trajectories (Table S1), resulting gene lists were used as input for computing overlap with the Molecular Signatures Database Hallmark gene set collection using the Gene Set Enrichment Analysis tool (Liberzon et al., 2015; Subramanian et al., 2005). Results were plotted in Figure S2E using the calculated FDR q-values, analog of hypergeometric p-value after correction for multiple hypothesis testing according to Benjamini and Hochberg (Subramanian et al., 2005).

#### Analysis of Embryonic Chimeras

***Differential abundance testing*** was performed using edgeR (McCarthy et al., 2012). Each 10× sample was considered as a replicate, and mapped cell type counts were used in place of gene counts. A separate linear model was fitted for E7.5 and E8.5 chimeras. Each linear model contained an intercept value specific to each biological replicate (i.e., pools of chimaeric embryos – one sample tdTom^+^ and the other tdTom^-^). A factor term was included for the injected samples from the WT chimeras, and another was included for the injected samples from the *T*^-/-^ chimeras. Differential abundance was tested using the contrast between these two factor terms, effectively asking whether the injected cell type frequency differed between the WT and *T*^-/-^ chimeras. This approach is preferable to testing entirely within the *T*^-/-^ chimeras, where the tdTom^-^ fraction of cells may be influenced by aberrant behaviour of the *T*^-/-^ cells. The intra-chimera approach is also vulnerable to confounding injected status (which may subtly affect cell behaviours) with genotype; the inter-chimera approach is not confounded. The use of wild-type chimeras also allows incorporation of the intrinsic variability of a mutation-free chimera system into the model. Finally, the use of edgeR allows sharing of uncertainty estimates across cell types with similar frequency in this sample-limited experiment. edgeR models were fitted and contrasts tested using the functions calcNormFactors, glmQLFit, and glmQLFTest.

#### Differential Expression Analyses

Differential expression testing was performed using the scran function findMarkers using default parameters. There was one exception. For the across-background NMP differential expression (Figure 6A), cells were selected with DPT values between 1.25 and 1.6. However, different distributions of cells along this section could induce apparent differential expression due to positions along ordering, rather than due to differences in genetic background. Here, we used the more sophisticated edgeR model, where we also fit the centred DPT values as a model coefficient to control for different distributions along the cell ordering. For this model, we tested against an absolute log_2_ fold-change of 0.5 as the edgeR model proved extremely sensitive to very small differences in expression level.

#### Relative Ratio Comparisons

In Figures 5A and 5B, relative contribution of injected cells to NMPs vs Posterior somites trajectories are calculated in E8.5 and E7.5 embryonic chimeras, respectively. Each point corresponds to an independent experiment (pool of chimaeric embryos), and calculated as: relative ratio = (number of tdTom^+^ on NMPs trajectory / number of tdTom^-^ on NMPs trajectory) / (number of tdTom^+^ on posterior somites trajectory / number of tdTom^-^ on posterior somites trajectory). This approach is robust to chimera-wide composition effects, as cell numbers are normalised using the host cells from each sample. To assess the difference in ratios between chimera types (i.e. WT into WT vs *T*^-/-^ into WT chimeras). p-values were estimated from 1000 permutations of the cells' trajectory labels.

#### Quantification of Primordial Germ Cells

Differences in Alkaline Phosphatase-positive PGC counts in *T*-expressing vs *T*^-/-^ mouse embryos at the headfold stage were assessed using an unpaired two-sample t-test (Figure S4J).

### Additional Resources

The code used to perform these analyses is available at https://github.com/MarioniLab/TChimeras2020. A singularity image that contains the exact versions of software used can be downloaded from the Github repository. An online visualisation tool is available at https://marionilab.cruk.cam.ac.uk/EarlySomites2020/.

## Supplemental Information

Document S1. Figures S1–S6

Table S1. Comparison between Transcriptional Trajectories, Related to Figure 2 Results of trajectory comparison for variable genes across trajectories. Lower p and FDR values correspond to more distinct expression patterns over time, genes with higher p and FDR values have more similar expression patterns. See also STAR Methods.

Table S2. Differential Expression Analysis between Cells Biased toward Anterior and Posterior Somitic Tissues, Related to Figures 3 and S3 Differential expression analysis results comparing cells biased toward Posterior somitic tissues vs cells biased toward Anterior somitic tissues at E7.5 and E7.0.

Table S3. Genes Spatially Enriched in Anterior and Posterior Mesoderm, Related to Figures 3 and S3 Genes enriched in the anterior and posterior mesoderm of the Peng et al. (2019) dataset; lists resulting from the “Gene Search by Pattern” tool provided by the eGastrulation database (see STAR Methods), and used as input for *Z* score calculations in Figure S3C.

Table S4. Differential Expression Analysis between Cells Biasedd toward NMPs and Somitic Tissues, Related to Figure 3 Differential expression analysis results comparing cells biased toward NMP vs cells biased toward somitic tissues at E7.5.

Table S5. Transcriptional Effects of T Knockout in E8.5 NMPs, Related to Figure 6 Differential expression analysis results comparing cells tdTom^+^ vs tdTom^−^ cells within the overrepresented NMP subset in E8.5 *T*^−/−^ chimeras.

Table S6. Transcriptional Effects of T Knockout at E7.5, Related to Figure 6 Differential expression analysis results comparing tdTom^+^ versus tdTom^−^ cells within the E7.5 cell subsets biased toward Anterior somitic tissues, Posterior somitic tissues and NMP *T*^−/−^ chimeras and WT control chimeras.

Document S2. Article plus Supplemental Information
