# Supplementary material for: Diverse Routes toward Early Somites in the Mouse Embryo
Source: Dev Cell. 2021 Jan 11;56(1):141–153.e6. doi: 10.1016/j.devcel.2020.11.013 (PMC7808755; doi:10.1016/j.devcel.2020.11.013)
Supplement: Document S1. Figures S1–S6 [file mmc1.pdf]

Developmental Cell, Volume *56*

Supplemental Information

Diverse Routes toward Early Somites

in the Mouse Embryo

Carolina Guibentif, Jonathan A. Griffiths, Ivan Imaz-Rosshandler, Shila Ghazanfar, Jennifer Nichols, Valerie Wilson, Berthold Göttgens, and John C. Marioni

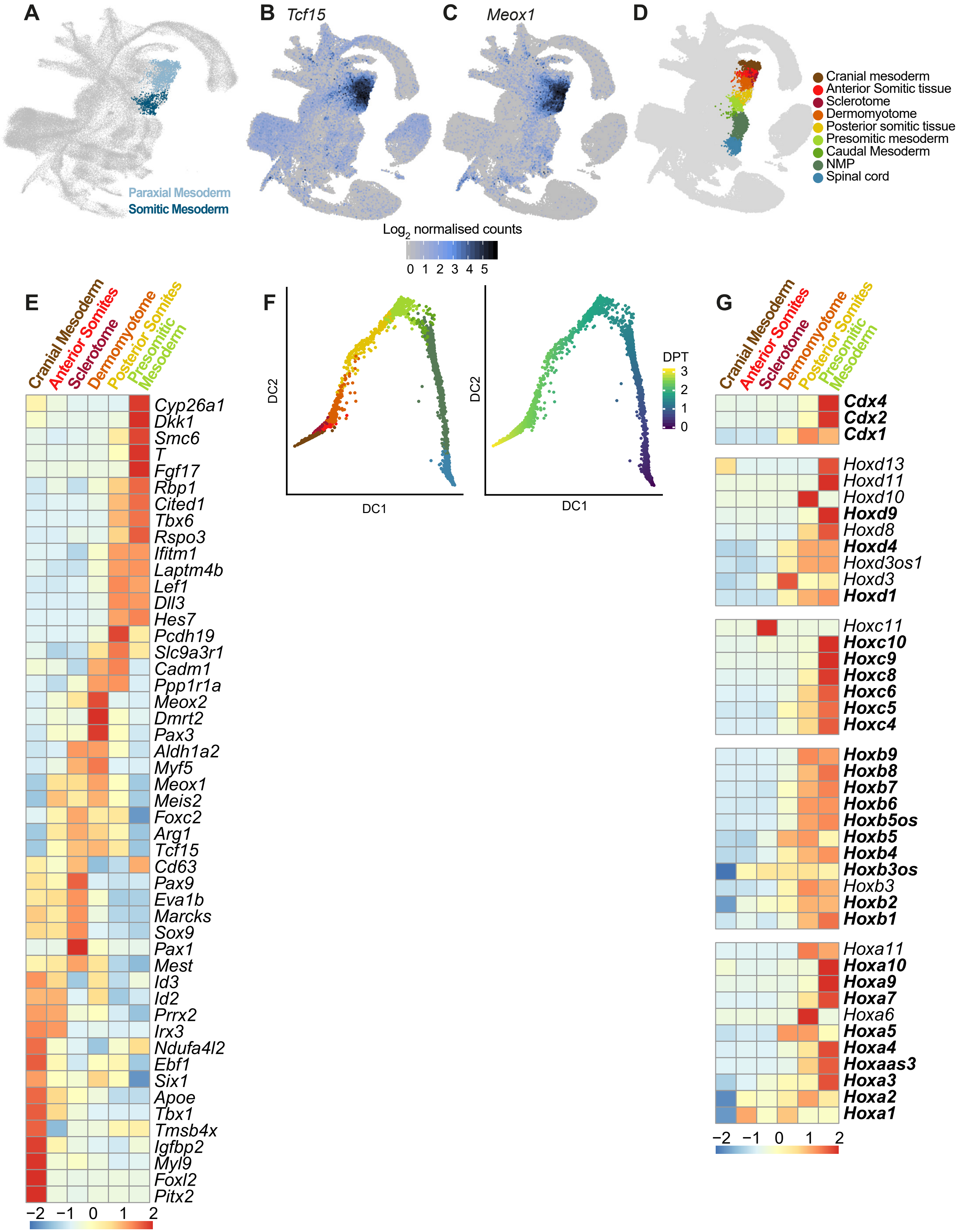

A
Paraxial Mesoderm
Somitic Mesoderm
B Tcf15
C Meox1
D
Cranial mesoderm
Anterior Somitic tissue
Sclerotome
Dermomyotome
Posterior somitic tissue
Presomitic mesoderm
Caudal Mesoderm
NMP
Spinal cord
Log2 normalised counts
0 1 2 3 4 5
E
Cranial Mesoderm
Anterior Somites
Sclerotome
Dermomyotome
Posterior Somites
Presomitic Mesoderm
Cyp26a1
Dkk1
Smc6
T
Fgf17
Rbp1
Cited1
Tbx6
Rspo3
Ifitm1
Laptm4b
Lef1
Dll3
Hes7
Pcdh19
Slc9a3r1
Cadm1
Ppp1r1a
Meox2
Dmrt2
Pax3
Aldh1a2
Myf5
Meox1
Meis2
Foxc2
Arg1
Tcf15
Cd63
Pax9
Eva1b
Marcks
Sox9
Pax1
Mest
Id3
Id2
Prrx2
Irx3
Ndufa4l2
Ebf1
Six1
Apoe
Tbx1
Tmsb4x
Igfbp2
Myl9
Foxl2
Pitx2
−2 −1 0 1 2
F
DC2
DC1
DPT
3
2
1
0
G
Cranial Mesoderm
Anterior Somites
Sclerotome
Dermomyotome
Posterior Somites
Presomitic Mesoderm
Cdx4
Cdx2
Cdx1
Hoxd13
Hoxd11
Hoxd10
Hoxd9
Hoxd8
Hoxd4
Hoxd3os1
Hoxd3
Hoxd1
Hoxc11
Hoxc10
Hoxc9
Hoxc8
Hoxc6
Hoxc5
Hoxc4
Hoxb9
Hoxb8
Hoxb7
Hoxb6
Hoxb5os
Hoxb5
Hoxb4
Hoxb3os
Hoxb3
Hoxb2
Hoxb1
Hoxa11
Hoxa10
Hoxa9
Hoxa7
Hoxa6
Hoxa5
Hoxa4
Hoxaas3
Hoxa3
Hoxa2
Hoxa1
−2 −1 0 1 2

Figure S1. Transcriptional characterization of axis elongation-related tissues (related to Figure 1):

(A) UMAP layout from Pijuan-Sala et al. (2019), highlighting clusters containing paraxial cell types used for further subclustering.
(B) Expression levels of *Tcf15* in the Atlas layout.
(C) Expression levels of *Meox1* in the Atlas layout.
(D) Atlas layout highlighting the newly defined axial elongation-related tissues present at E8.5
(E) Centred and scaled average expression levels of signature genes characterizing the newly identified elongation-related tissues present at E8.5.
(F) Left: diffusion map of elongation-related tissues present at E8.5 coloured by newly-identified subcluster (see corresponding legend in (D)); right: diffusion map of elongation-related tissues present at E8.5 colored by Diffusion Pseudotime value (i.e. position in the one-dimensional ordering).
(G) Centred and scaled average expression levels of Cdx and Hox transcription factors in the newly identified elongation-related tissues present at E8.5. Genes differentially expressed with FDR < 0.05 in at least one comparison are highlighted in bold font.

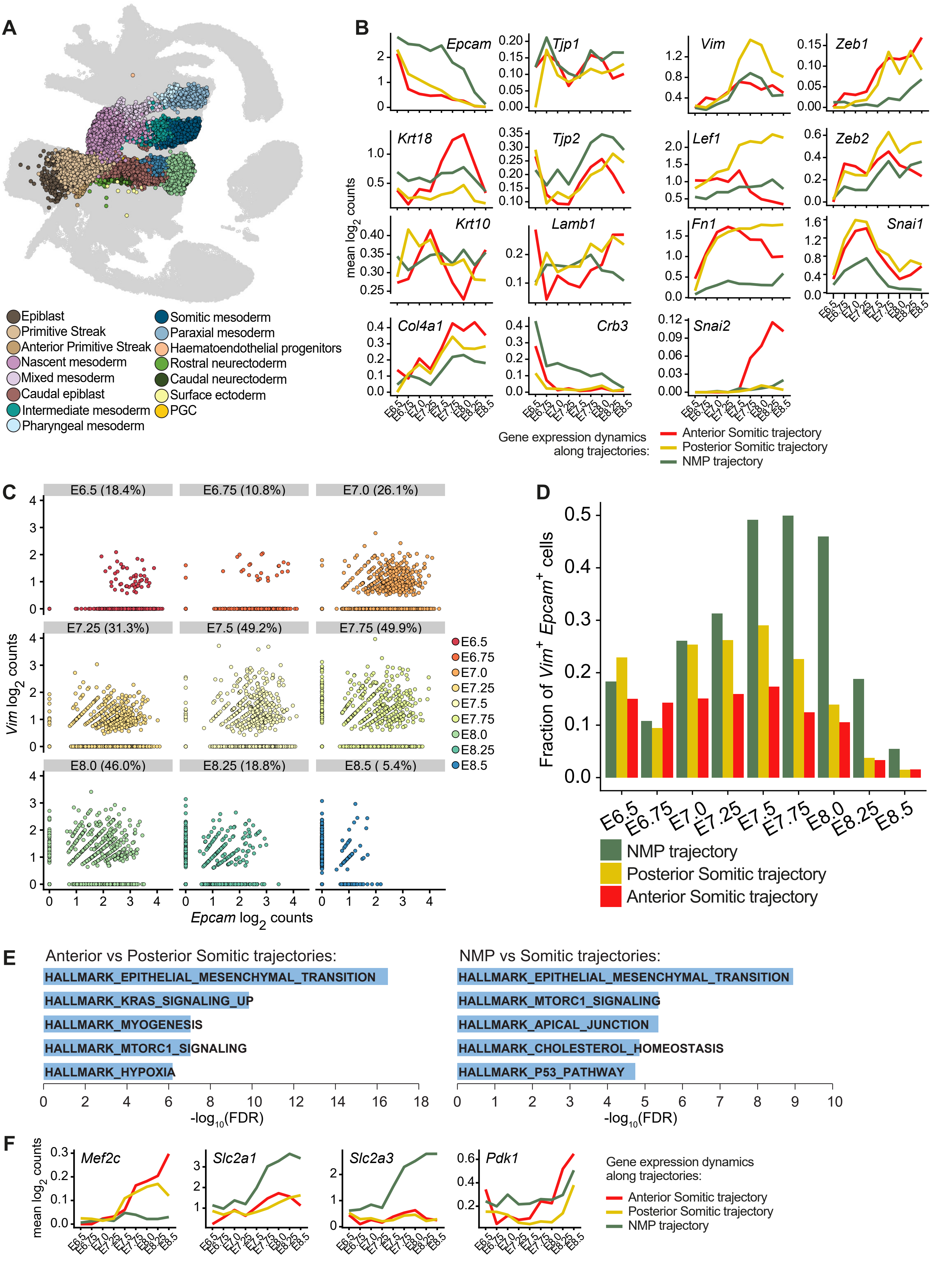

A
Epiblast
Primitive Streak
Anterior Primitive Streak
Nascent mesoderm
Mixed mesoderm
Caudal epiblast
Intermediate mesoderm
Pharyngeal mesoderm
Somitic mesoderm
Paraxial mesoderm
Haematoendothelial progenitors
Rostral neurectoderm
Caudal neurectoderm
Surface ectoderm
PGC
B
Epcam
Tjp1
Vim
Zeb1
Krt18
Tjp2
Lef1
Zeb2
Krt10
Lamb1
Fn1
Snai1
Col4a1
Crb3
Snai2
mean log2 counts
Gene expression dynamics along trajectories:
Anterior Somitic trajectory
Posterior Somitic trajectory
NMP trajectory
C
E6.5 (18.4%)
E6.75 (10.8%)
E7.0 (26.1%)
E7.25 (31.3%)
E7.5 (49.2%)
E7.75 (49.9%)
E8.0 (46.0%)
E8.25 (18.8%)
E8.5 (5.4%)
Vim log2 counts
Epcam log2 counts
E6.5
E6.75
E7.0
E7.25
E7.5
E7.75
E8.0
E8.25
E8.5
D
Fraction of Vim+ Epcam+ cells
NMP trajectory
Posterior Somitic trajectory
Anterior Somitic trajectory
E
Anterior vs Posterior Somitic trajectories:
HALLMARK_EPITHELIAL_MESENCHYMAL_TRANSITION
HALLMARK_KRAS_SIGNALING_UP
HALLMARK_MYOGENESIS
HALLMARK_MTORC1_SIGNALING
HALLMARK_HYPOXIA
-log10(FDR)
NMP vs Somitic trajectories:
HALLMARK_EPITHELIAL_MESENCHYMAL_TRANSITION
HALLMARK_MTORC1_SIGNALING
HALLMARK_APICAL_JUNCTION
HALLMARK_CHOLESTEROL_HOMEOSTASIS
HALLMARK_P53_PATHWAY
-log10(FDR)
F
Mef2c
Slc2a1
Slc2a3
Pdk1
mean log2 counts
Gene expression dynamics along trajectories:
Anterior Somitic trajectory
Posterior Somitic trajectory
NMP trajectory

Figure S2. Multiple transcriptional trajectories towards somites (related to Figure 2):

(A) Pijuan-Sala et al. (2019) annotation of cells belonging to the developmental trajectories for NMPs as well as for anterior vs posterior somites present at E8.5, predicted using Waddington Optimal Transport analysis (See Figure 2A, B).
(B) Expression dynamics of epithelial and mesenchymal signature genes along the three developmental trajectories. y-axis: mean $\log_2$(normalised counts).
(C) Scatterplots of log2(normalised counts) for *Epcam* (epithelial marker) vs *Vim* (mesenchymal marker) in NMP-fated cells along developmental time-points suggest a state of incomplete EMT. Titles indicate percentage of cells in which both genes were detected.
(D) Quantification of *Epcam/Vim* double-positive cells in transcriptional trajectories towards NMP, Anterior Somitic tissues and Posterior somitic tissues throughout development suggest a high incidence of cells in a incomplete EMT state on the NMP trajectory.
(E) Top 5 Hallmark signatures resulting from Gene Set Enrichment Analysis with (left) the genes displaying different behaviours over time in the anterior and posterior somitic trajectories, and (right) the genes with expression dynamics specific to the NMP trajectory (these are genes overlapping between comparisons: NMP vs anterior somitic trajectories and NMP vs posterior somitic trajectories). See also Supplemental Table 1 and Methods.
(F) Expression dynamics along the three developmental trajectories for genes featured in the “Myogenesis” and “mTorC1 signaling” Hallmark signatures in (E).

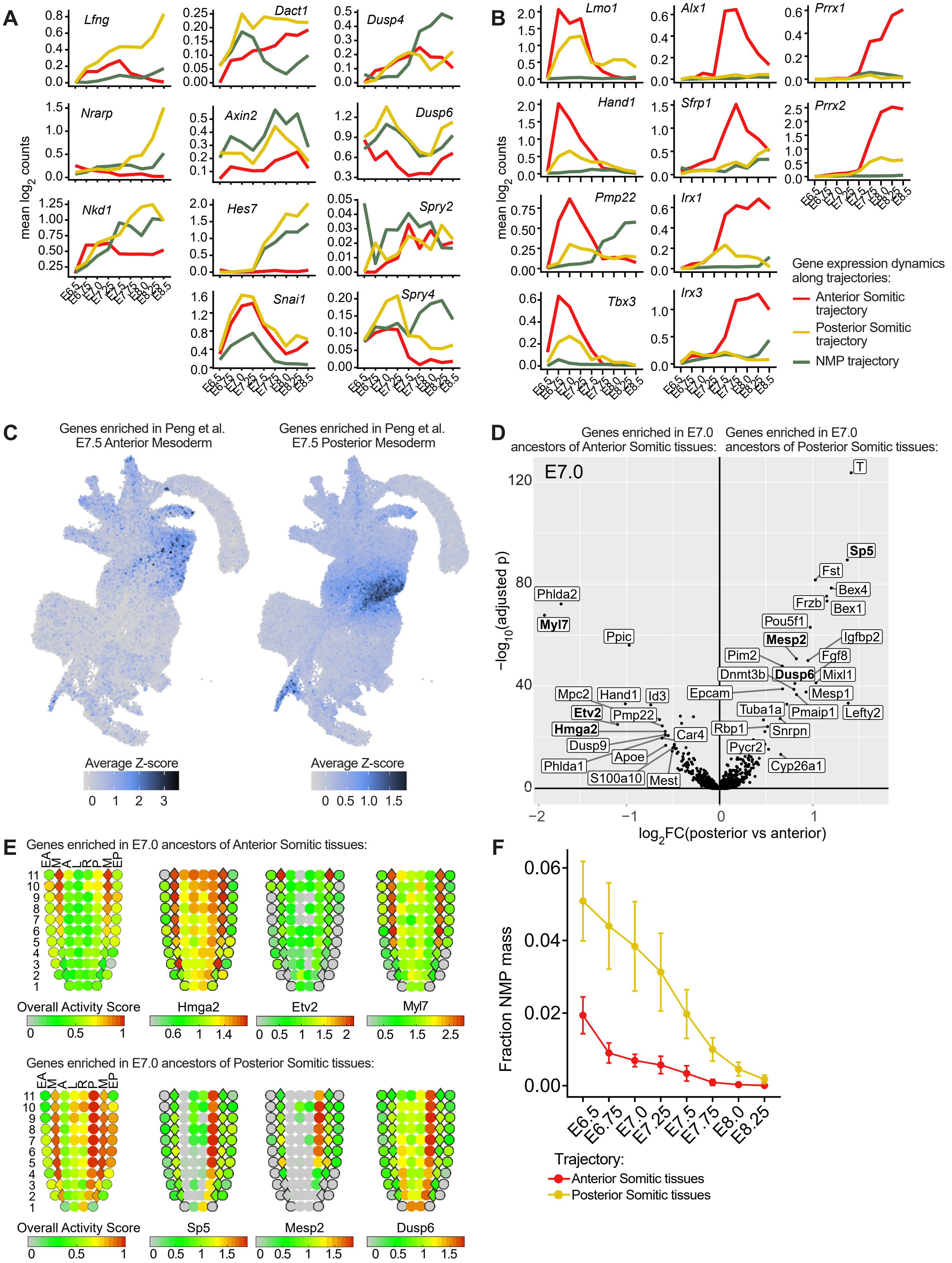

A
Lfng
Dact1
Dusp4
Nrarp
Axin2
Dusp6
Nkd1
Hes7
Spry2
Snai1
Spry4
mean log2 counts
E6.5
E6.75
E7.0
E7.25
E7.5
E7.75
E8.0
E8.25
E8.5
B
Lmo1
Alx1
Prrx1
Hand1
Sfrp1
Prrx2
Pmp22
Irx1
Tbx3
Irx3
Gene expression dynamics along trajectories:
Anterior Somitic trajectory
Posterior Somitic trajectory
NMP trajectory
C
Genes enriched in Peng et al. E7.5 Anterior Mesoderm
Genes enriched in Peng et al. E7.5 Posterior Mesoderm
Average Z-score
D
Genes enriched in E7.0 ancestors of Anterior Somitic tissues:
Genes enriched in E7.0 ancestors of Posterior Somitic tissues:
E7.0
−log10(adjusted p)
log2FC(posterior vs anterior)
T
Sp5
Fst
Bex4
Frzb
Bex1
Pou5f1
Mesp2
Igfbp2
Pim2
Fgf8
Dnmt3b
Dusp6
Mixl1
Epcam
Mesp1
Tuba1a
Pmaip1
Lefty2
Rbp1
Snrpn
Pycr2
Cyp26a1
Phlda2
Myl7
Ppic
Mpc2
Hand1
Id3
Etv2
Pmp22
Hmga2
Car4
Dusp9
Apoe
Phlda1
S100a10
Mest
E
Genes enriched in E7.0 ancestors of Anterior Somitic tissues:
EA
M
A
L
R
P
M
EP
Overall Activity Score
Hmga2
Etv2
Myl7
Genes enriched in E7.0 ancestors of Posterior Somitic tissues:
Overall Activity Score
Sp5
Mesp2
Dusp6
F
Fraction NMP mass
Trajectory:
Anterior Somitic tissues
Posterior Somitic tissues

Figure S3. Spatial segregation of somitic developmental trajectories (related to Figure 3):

(A) Expression dynamics of markers of the clock and wave-front somitogenesis model along the three developmental trajectories. y-axis: mean log_2(normalised counts).
(B) Gene expression dynamics of candidate regulators specific to the anterior somite development along the three developmental trajectories. y-axis: mean log_2(normalised counts).
(C) Levels of average Z-scores for the genesets retrieved from Peng et al. (2019), as enriched in E7.5 anterior mesoderm (left) and in E7.5 posterior mesoderm (right), in the Pijuan-Sala et al. (2019) dataset. To compare with Figure 2A. See also Supplemental Table 3 and Methods.
(D) Differential expression analysis between E7.0 cells present on the posterior somitic tissues trajectory vs E7.0 cells on the anterior somitic tissue trajectory.
(E) Schematics of the overall "activity score" for the genes significantly differentially expressed in each trajectory (from analysis in D), calculated using the eGastrulation tool for E7.0 spatial data (Peng et al., 2019) and expression levels in log_{10}(FPKM+1) for selected genes highlighted in bold font in (D). See also legend for Figure 3B. M: whole Mesoderm; L: Left lateral; R: Right lateral.
(F) Average fraction of optimal transport mass for the NMP lineage, in cells allocated to the anterior or posterior somite trajectories. In all comparisons, cells on the posterior somitic tissue trajectory have significantly higher NMP mass (adjusted $p < 10^{-10}$, Student's t-test; error bars: standard deviation).

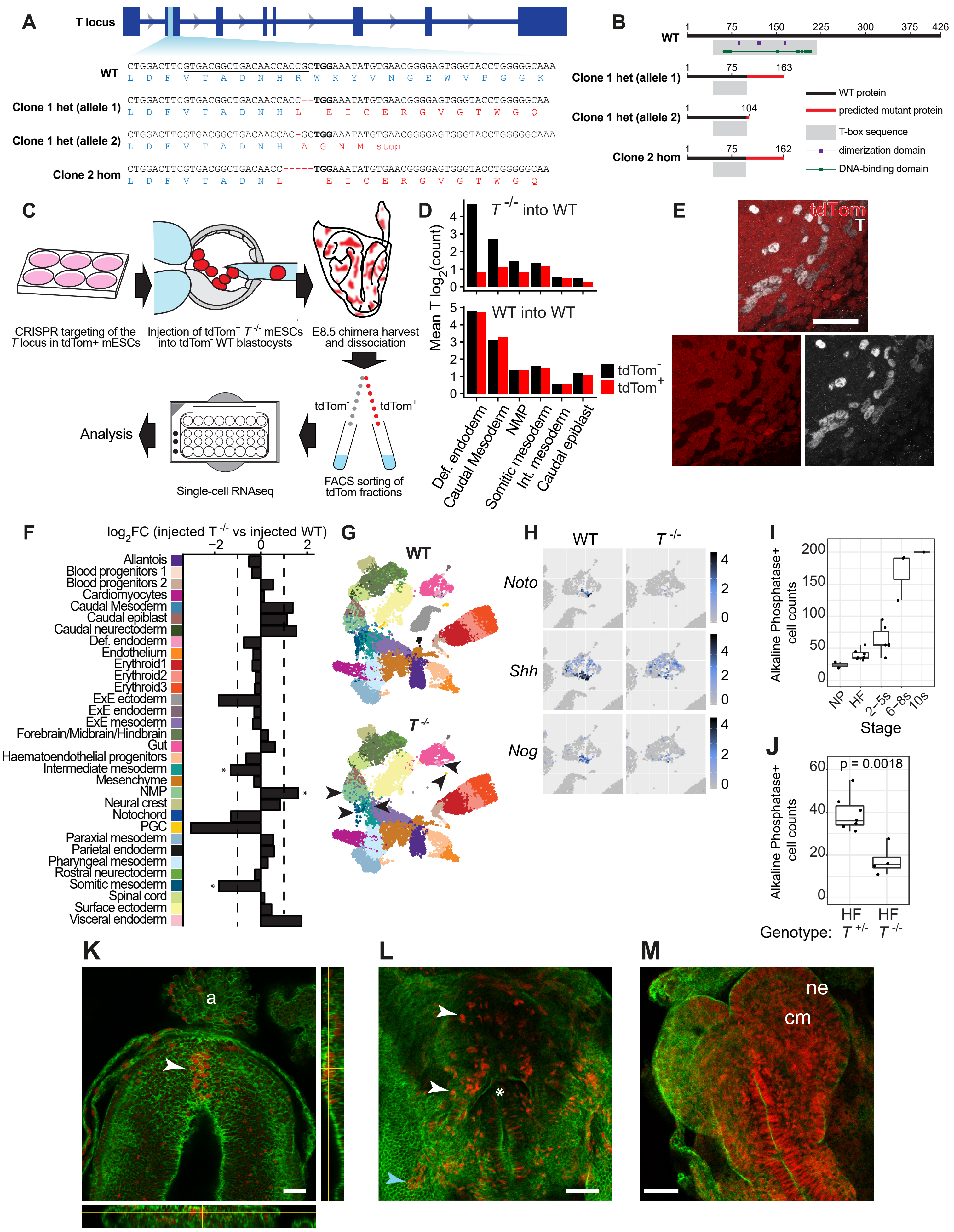

A
T locus
WT
Clone 1 het (allele 1)
Clone 1 het (allele 2)
Clone 2 hom
B
WT
Clone 1 het (allele 1)
Clone 1 het (allele 2)
Clone 2 hom
WT protein
predicted mutant protein
T-box sequence
dimerization domain
DNA-binding domain
C
CRISPR targeting of the T locus in tdTom+ mESCs
Injection of tdTom+ T -/- mESCs into tdTom- WT blastocysts
E8.5 chimera harvest and dissociation
tdTom-
tdTom+
FACS sorting of tdTom fractions
Single-cell RNAseq
Analysis
D
T -/- into WT
WT into WT
Mean T log2(count)
tdTom-
tdTom+
Def. endoderm
Caudal Mesoderm
NMP
Somitic mesoderm
Int. mesoderm
Caudal epiblast
E
tdTom
T
F
log2FC (injected T -/- vs injected WT)
Allantois
Blood progenitors 1
Blood progenitors 2
Cardiomyocytes
Caudal Mesoderm
Caudal epiblast
Caudal neurectoderm
Def. endoderm
Endothelium
Erythroid1
Erythroid2
Erythroid3
ExE ectoderm
ExE endoderm
ExE mesoderm
Forebrain/Midbrain/Hindbrain
Gut
Haematoendothelial progenitors
Intermediate mesoderm
Mesenchyme
NMP
Neural crest
Notochord
PGC
Paraxial mesoderm
Parietal endoderm
Pharyngeal mesoderm
Rostral neurectoderm
Somitic mesoderm
Spinal cord
Surface ectoderm
Visceral endoderm
G
WT
T -/-
H
WT
T -/-
Noto
Shh
Nog
I
Alkaline Phosphatase+ cell counts
NP
HF
2–5s
6–8s
10s
Stage
J
p = 0.0018
Alkaline Phosphatase+ cell counts
HF
HF
Genotype: T +/- T -/-
K
a
L
M
ne
cm

Figure S4. Dissecting the cell-autonomous role of *T* (related to Figure 4):

(A) Schematic of sequencing results of the CRISPR-targeting of *T* locus showing frameshift mutations in both analyzed clones. Het: heterozygous; hom: homozygous.
(B) Schematics of the predicted protein sequences resulting from mutations depicted in (A). All detected mutations result in an early stop codon and prevent the formation of DNA-binding and dimerization domains, precluding the existence of a functional T protein.
(C) Schematic of experimental strategy for generation of E8.5 $T^{-/-}$ chimeras.
(D) Average detected levels of *T* transcript in *T*-expressing cell types in $T^{-/-}$ (top) and WT control (bottom) chimeras.
(E) Confocal image of a $T^{-/-}$ chimeric embryo with high chimerism levels showing negative correlation between tdTomato and T protein. Scale bar: 50µm.
(F) Differential abundance testing of embryonic cell types in $T^{-/-}$ chimeras compared to the WT chimera controls. Only cell types comprising more than 10 chimeric cells are displayed (excluded cell types: Mixed Mesoderm and Epiblast containing 3 and 1 cells respectively). *: BH-corrected $p < 0.1$, $n = 4$ independent experiments.
(G) UMAP representation of the scRNAseq data of chimeric embryos, split by genotype. See (F) for colour code of the different cell types. Arrowheads highlight the cell types with observed significant increased or decreased representation in the $T^{-/-}$ cell fraction as depicted in Figure 4A, corresponding to NMPs, somitic mesoderm, Intermediate mesoderm. Notochord and PGCs (depleted but below statistical significance) are also highlighted.
(H) Close-up of the definitive endoderm and notochord lineages in the UMAP in (G), displaying expression levels for key notochord genes dysregulated in the $T^{-/-}$ fraction. Colour scale represents Log_2(normalized counts) for each transcript.
(I) Counts of Alkaline Phosphatase-positive PGCs in T-expressing mouse embryos increase exponentially throughout development. NP: Neural Plate (E7.5); HF: Headfold (E7.75); s: somite stage.
(J) Counts of Alkaline Phosphatase-positive PGCs in T-expressing and $T^{-/-}$ mouse embryos at the headfold stage showing a significant decrease in numbers in homozygous mutant embryos (p-value = 0.0018, Student's t-test).
(K) Confocal image of the caudal region of an E8.5 chimeric embryo with low chimerism showing accumulation of $tdTom^{+}$ cells within the remnant primitive streak region (arrowhead). a: allantois, containing $tdTom^{+}$ cells. Scale bar: 50µm.
(L) Confocal image of the heart region of an E8.5 chimeric embryo showing contribution of $tdTom^{+}$ cells to cardiac tissue (white arrowheads), foregut diverticulum (asterisk) and extra-embryonic mesoderm (blue arrowhead). Scale bar: 100µm.
(M) Confocal image of the cranial region of an E8.5 chimeric embryo with high chimerism, showing contribution of $tdTom^{+}$ cells to cranial tissues, including cranial mesenchyme (cm) and neurectoderm (ne). Scale bar: 100µm.

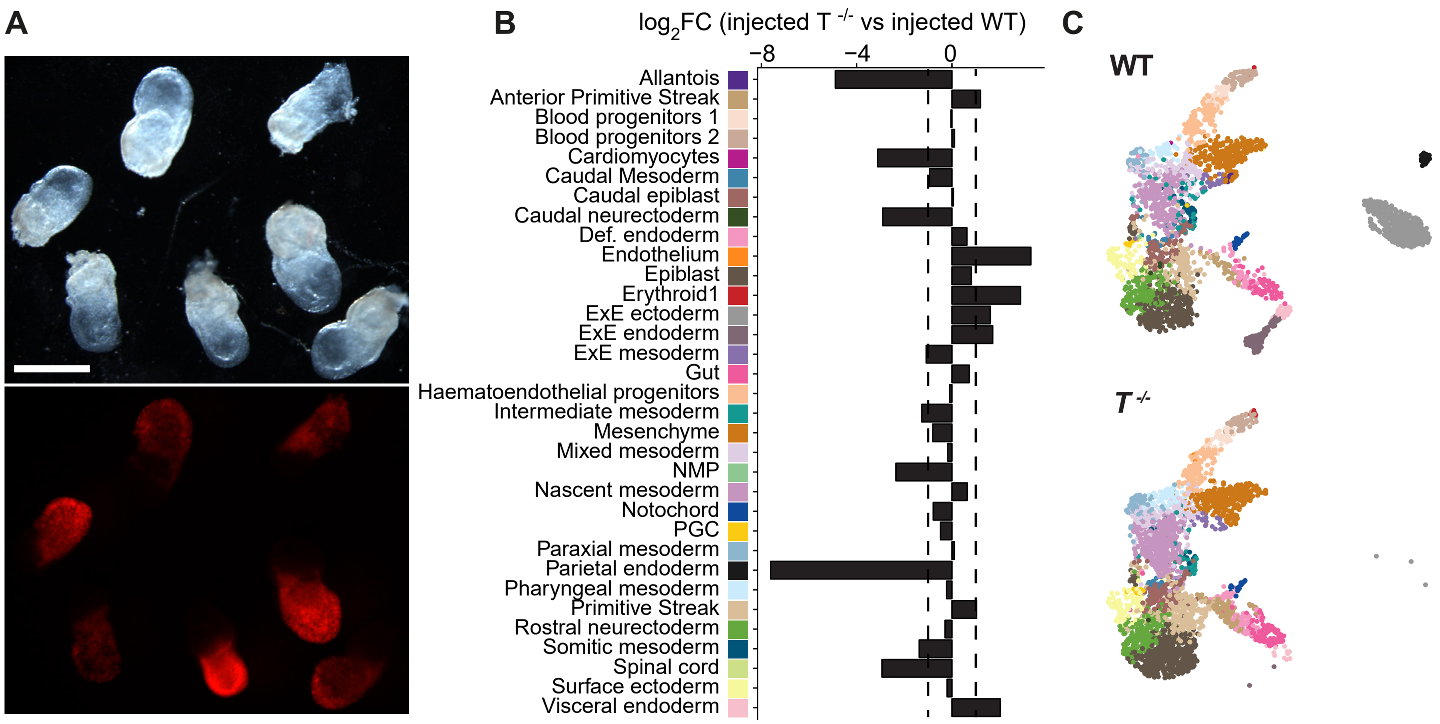

Figure S5. Lineage contribution of $T^{-/-}$ cells in E7.5 chimaeras (related to Figure 5):

(A) Representative images of E7.5 $T^{-/-}$ chimeric embryos, bright field (top) and tdTomato fluorescence (bottom). Scale bar: 500µm.
(B) Differential abundance testing of embryonic cell types in E7.5 $T^{-/-}$ chimeras compared to the WT chimera controls. No statistically significant deviations in relative abundance were observed (n = 3 independent experiments).
(C) UMAP representation of the scRNAseq data of chimeric embryos, split by genotype. See (B) for colour code of the different cell types.

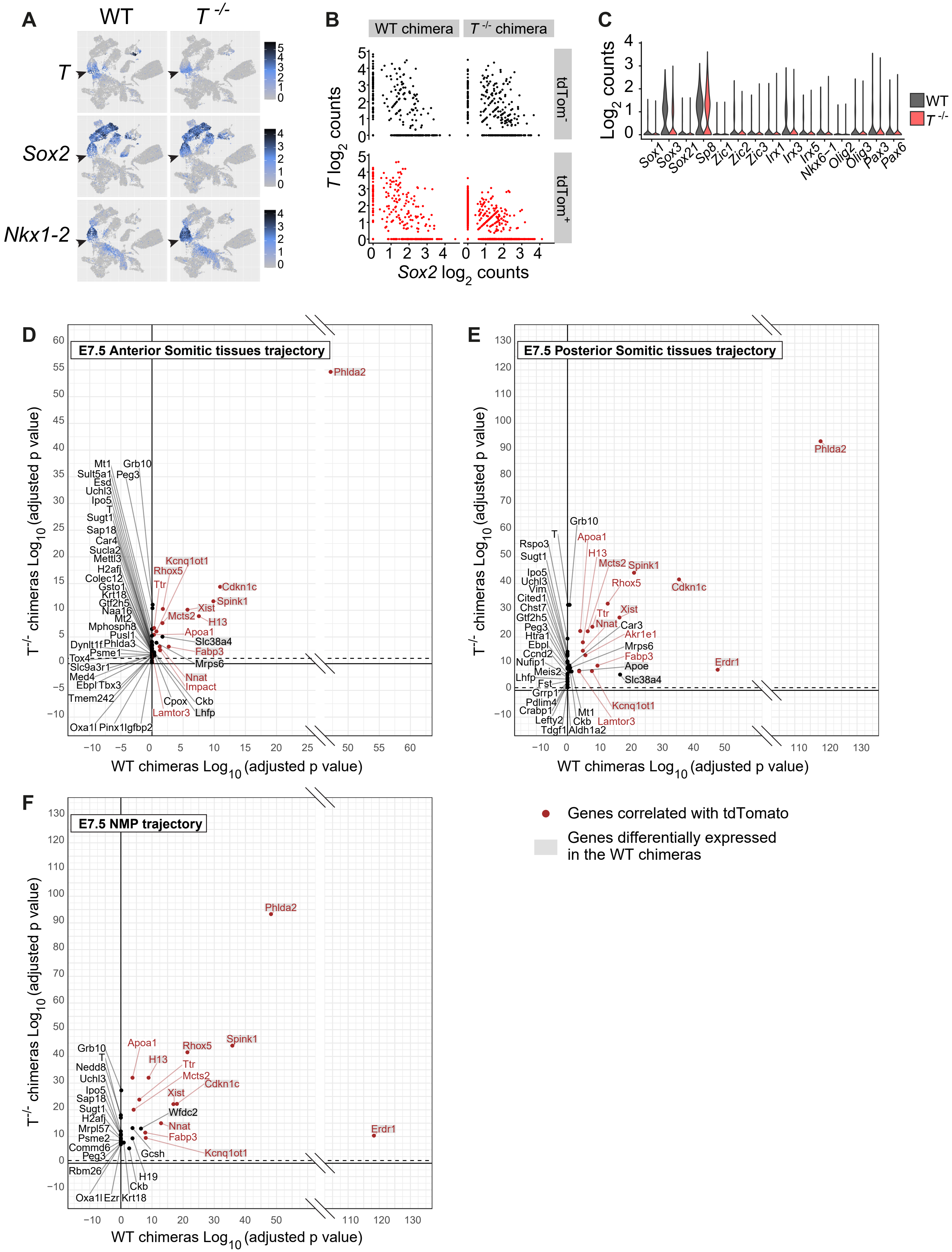
A
WT
T -/-
T
Sox2
Nkx1-2
B
WT chimera
T -/- chimera
tdTom-
tdTom+
T log2 counts
Sox2 log2 counts
C
Log2 counts
WT
T -/-
Sox1
Sox3
Sox21
Sp8
Zic1
Zic2
Zic3
Irx1
Irx3
Irx5
Nkx6-1
Olig2
Olig3
Pax3
Pax6
D
E7.5 Anterior Somitic tissues trajectory
Phlda2
Cdkn1c
Spink1
Kcnq1ot1
Rhox5
Ttr
Xist
H13
Mcts2
Apoa1
Fabp3
Slc38a4
Mrps6
Nnat
Impact
Cpox
Ckb
Lamtor3
Lhfp
Mt1
Grb10
Sult5a1
Peg3
Esd
Uchl3
Ipo5
T
Sugt1
Sap18
Car4
Sucla2
Mettl3
H2afj
Colec12
Gsto1
Krt18
Gtf2h5
Naa16
Mt2
Mphosph8
Pusl1
Dynlt1f
Phlda3
Psme1
Tox4
Slc9a3r1
Med4
Ebpl
Tbx3
Tmem242
Oxa1l
Pinx1
Igfbp2
T-/- chimeras Log10 (adjusted p value)
WT chimeras Log10 (adjusted p value)
E
E7.5 Posterior Somitic tissues trajectory
Phlda2
Cdkn1c
Erdr1
Grb10
T
Apoa1
Rspo3
Sugt1
H13
Mcts2
Spink1
Rhox5
Ipo5
Uchl3
Vim
Cited1
Chst7
Gtf2h5
Peg3
Htra1
Ebpl
Ccnd2
Nufip1
Meis2
Lhfp
Fst
Grrp1
Pdlim4
Crabp1
Lefty2
Tdgf1
Ttr
Xist
Nnat
Car3
Akr1e1
Mrps6
Fabp3
Apoe
Slc38a4
Kcnq1ot1
Mt1
Ckb
Lamtor3
Aldh1a2
T-/- chimeras Log10 (adjusted p value)
WT chimeras Log10 (adjusted p value)
F
E7.5 NMP trajectory
Phlda2
Spink1
Rhox5
Apoa1
H13
Ttr
Mcts2
Cdkn1c
Xist
Wfdc2
Nnat
Fabp3
Kcnq1ot1
Erdr1
Gcsh
Grb10
T
Nedd8
Uchl3
Ipo5
Sap18
Sugt1
H2afj
Mrpl57
Psme2
Commd6
Peg3
Rbm26
H19
Ckb
Oxa1l
Ezr
Krt18
T-/- chimeras Log10 (adjusted p value)
WT chimeras Log10 (adjusted p value)
Genes correlated with tdTomato
Genes differentially expressed in the WT chimeras

Figure S6. Molecular analysis of $T^{-/-}$ chimaeras (related to Figure 6):

(A) UMAPs of E8.5 $T^{-/-}$ chimera cells, split by genotype with expression levels of NMP marker genes. Arrowhead: NMP cells. See also Figure S4G.
(B) Scatterplots of $\log_2$(normalised counts) for *T* and *Sox2* in single E8.5 NMP-mapped cells in WT into WT chimeras (left) and $T^{-/-}$ chimeras (right). Although *T* and *Sox2* appear generally anti-correlated, *Sox2* is not up-regulated in the absence of T (lower-right), arguing against cross-antagonism of these two regulators in NMPs.
(C) Violin plots of genes featuring a neural signature in $T^{-/-}$ chimera NMP-mapped cells, showing no up-regulation in the $T^{-/-}$ fraction.
(D-F) Genes significantly deregulated in E7.5 $T^{-/-}$ chimera cells mapping to anterior somitic tissues (D), posterior somitic tissues (E), NMP (F) trajectories plotted by adjusted p values corresponding to expression levels comparison of tdTom^{+} injected cells and tdTom^{-} host cells in WT (x-axis) and $T^{-/-}$ chimeras (y-axis). Highlighted are genes excluded from Figure 6b analysis due to significant deregulation in WT chimeras (plotted in grey) or high correlation with the tdTomato transcript (plotted in brown) over whole WT chimeras. These highlighted genes are likely technical artefacts of the chimera generation procedure (e.g. imprinted genes).
